# Valorizing Duck Bone into Regenerable Hydroxyapatite Biochar: Pyrolysis Effects on Divalent Heavy Metal Adsorption Mechanisms

**DOI:** 10.3390/ijms27219991

**Published:** 2026-11-07

**Authors:** Sukanya Hongthong, Torpong Kreethachat, Saksit Imman, Nopparat Suriyachai, Anusorn Boonpoke, Kowit Suwannahong, Pattaranun Maskhunthod, Surachai Wongcharee

**Affiliations:** 1 Department of Mechanical Engineering, Faculty of Arts and Science, Chaiyaphum Rajabhat University, Chaiyaphum 36000, Thailand; sukanya.ho@cpru.ac.th; 2 School of Energy and Environment, University of Phayao, Phayao 56000, Thailand; torpong.kr@up.ac.th (T.K.); saksit.im@up.ac.th (S.I.); nopparat.su@up.ac.th (N.S.); iamanusorn@gmail.com (A.B.); 3 Department of Environmental Health, Faculty of Public Health, Burapha University, Chonburi 20131, Thailand; kowit.su@go.buu.ac.th; 4 Field of Environmental Engineering, Faculty of Engineering, Mahasarakham University, Mahasarakham 44150, Thailand; coke.wongcharee@gmail.com

**Keywords:** duck bone char, hydroxyapatite, adsorption, pyrolysis temperature, regeneration kinetics, circular economy

## Abstract

Duck bone, an under-used by-product of Southeast Asian poultry processing, was converted into hydroxyapatite-based bone char by single-step, reagent-free pyrolysis at 300, 400, and 500 °C and evaluated as a reusable Cu(II) adsorbent. Physical and chemical analyses showed progressive removal of the collagen–lipid matrix and preservation of a hydroxyapatite scaffold (98–99 wt% of the crystalline phases by Rietveld refinement, with minor calcite; apparent crystallite size ~7–8 nm across and ~23–25 nm along the c-axis). Surface area increased progressively from 33.0 to 84.2 m^2^ g^−1^ with pyrolysis temperature, whereas the Toth capacity increased steadily from 44.7 to 48.6 mg g^−1^, indicating that uptake is governed mainly by apatite-site accessibility rather than scaling proportionally with surface area. Sigmoidal Avrami kinetics with exponents of 1.25–1.48, sharply saturating isotherms, an endothermic enthalpy of 71–90 kJ mol^−1^, and points of zero charge of about pH 7–9.5 supported cooperative binding through Ca^2+^–Cu^2+^ ion exchange, phosphate and hydroxyl complexation, and proposed surface nucleation of Cu–phosphate clusters; Cu(OH)_2_ precipitation was not expected at the pH used. After five HCl regeneration cycles, DKB retained 73.1–80.0% of its initial efficiency, and the first-order decay constant decreased from 0.078 to 0.056 cycle^−1^ with increasing pyrolysis temperature. DKB produced at 500 °C combined the highest capacity, fastest kinetics, and greatest regeneration stability, establishing duck bone as a low-cost, circular feedstock for Cu(II) remediation.

## 1. Introduction

Copper (Cu) is an essential trace element, but it becomes a toxic pollutant when anthropogenic inputs raise its concentration in aquatic environments above natural background levels. The main anthropogenic sources are mining, metal manufacturing, fungicides, and brake pad wear, whereas natural sources include forest fires, volcanoes, and atmospheric dust [1,2]. Electroplating, electronics manufacturing, fertilizer, paper, and etching industries discharge Cu–bearing wastewater [3], and studies of rivers and basins show that Cu smelting and industrial discharges from cable, pigment, and mining industries have damaged the surrounding environment [4,5]. Because Cu^2+^ is non–degradable and bioaccumulative, it poses risks to the liver and kidneys and is listed as a priority heavy metal in wastewater discharge regulations [6].

Conventional treatments such as precipitation, ion exchange, membrane separation, electrowinning, and coagulation/flocculation each have drawbacks [7]. Precipitation and coagulation generate metal–laden sludge that requires further disposal, ion exchange consumes large amounts of reagents, and membranes, although highly effective, suffer from high cost, fouling, and energy demand [8,9]. Adsorption is therefore widely preferred for its simplicity, low cost, and high capacity. It also reduces sludge, allows metal recovery, and permits adsorbent regeneration, which most conventional technologies do not [8]. This has shifted research toward low-cost, eco-friendly adsorbents made from waste [7].

Biochar, a porous carbonaceous solid produced by the pyrolysis of biomass under oxygen-deficient conditions, is among the most promising low-cost adsorbents for heavy metals, with precipitation and ion exchange as the principal mechanisms [10]. Pyrolysis temperature is the most influential production parameter. Higher temperatures increase ash and mineral content, surface area, porosity, pore volume, and cation exchange capacity, while decreasing yield, moisture, and heteroatom (H, N, S, O) content [11]. Low-temperature chars remove inorganic contaminants mainly through electrostatic attraction and ion exchange, whereas high–temperature chars rely more on pore filling, hydrophobic interaction, and mineral precipitation [12]. Across many feedstocks, raising the pyrolysis temperature from 300 to 600 °C increases structural stability and heavy metal adsorption capacity [13].

Animal bone is a distinctive feedstock because its mineral fraction, calcium hydroxyapatite (Ca_10_(PO_4_)_6_(OH)_2_), provides metal-binding chemistry that lignocellulosic biochar lacks. Bone chars remove metals by electrostatic attraction, surface complexation, precipitation, and cation exchange [14], and hydroxyapatite adds co-precipitation and hydrogen-bond-assisted physical adsorption [15]. Cu(II) uptake on hydroxyapatite proceeds by surface complexation together with exchange for structural calcium, forming a Cu-substituted phase at the mineral surface rather than inside the lattice [16]. The World Health Organization recommends bone char for fluoride removal in low-income communities, and it is now also applied to heavy metals and organic pollutants [17]. Pig, cow, fish, and chicken bone chars have all been studied for Cu(II) or other heavy metals [18,19,20,21,22]. Duck bone char, however, is far less investigated than these other materials.

The poultry sector generates large and growing volumes of bone byproduct. Thailand’s broiler production rose by 4.5% in 2023, and global poultry meat consumption is projected to reach 181 million tons by 2025, creating a corresponding burden of bone, feather, and offal waste. Duck bone, a similarly abundant byproduct in Southeast Asian food markets, has received far less attention, and its temperature-resolved evaluation for Cu(II) removal has not been reported. Reusability is equally decisive. The economic and environmental case for a waste-derived adsorbent depends on the balance between synthesis cost, regeneration cost, and the wastewater generated during regeneration [23]. A material that performs well in one cycle but degrades quickly offers little advantage over disposable adsorbents, so multicycle tests, and not only initial capacity, should judge practical applicability.

This work therefore studies duck bone char (DKB) produced by slow pyrolysis at 300, 400, and 500 °C. Its objectives are to (1) prepare DKB at three pyrolysis temperatures; (2) characterize its physicochemical evolution by SEM–EDS, BET, CHNS, FTIR, and XRD; (3) evaluate Cu(II) adsorption through nonlinear kinetic, isotherm, and thermodynamic analysis, and (4) assess multi-cycle desorption–regeneration with a cycle–domain model that quantifies the rate of capacity loss. The novelty lies not in showing that bone char adsorbs Cu(II), which is already established for several bone-derived materials, but in the temperature-resolved integration of characterization, statistically selected adsorption models, mechanistic interpretation, and regeneration modeling. Together these establish a pyrolysis–structure–adsorption–regeneration relationship and a basis for judging both the initial uptake and the durability of duck bone-derived adsorbents.

## 2. Results and Discussion

### 2.1. Morphological Evolution of Duck Bone Char

**Figure 1:**
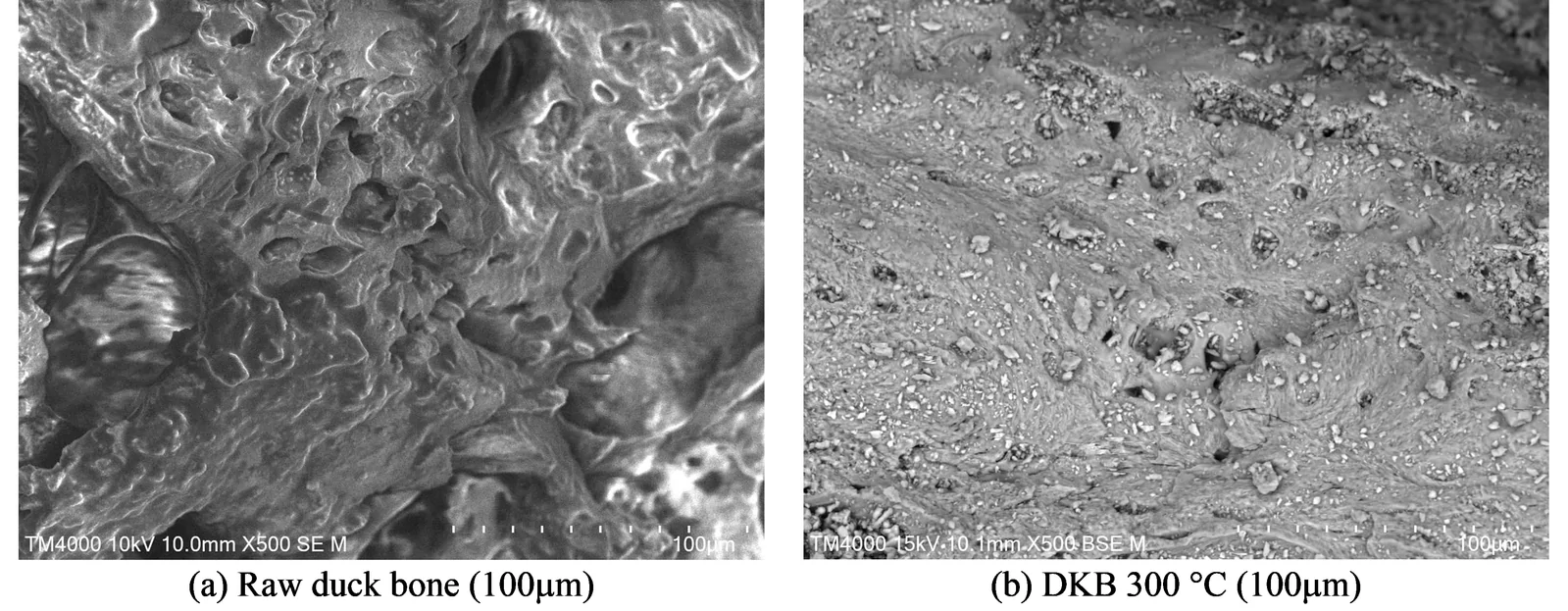

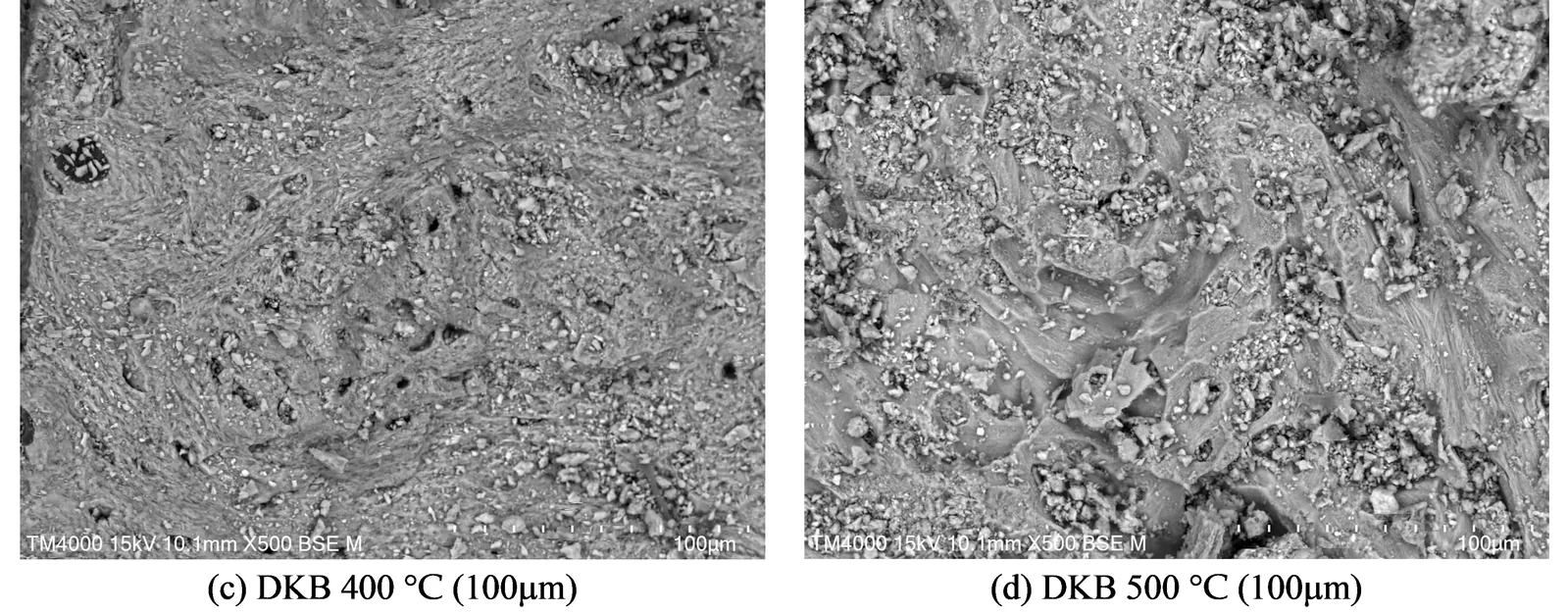
Morphological evolution of duck bone before and after pyrolysis: (**a**) SEM image of raw duck bone, and (**b**–**d**) DKB produced at 300, 400, and 500 °C, respectively.

SEM imaging shows the morphological evolution of the bone surface with pyrolysis temperature (Figure 1a–d). The raw, unpyrolyzed duck bone (Figure 1a) shows a ridged, fibrous surface topology with scattered pores and grooves, consistent with the collagen fiber bundles and vascular channels of cortical bone together with embedded mineral (hydroxyapatite) material. After pyrolysis at 300 °C (Figure 1b), the surface becomes finer-grained and more particulate, with small, mineral-rich grains distributed through the matrix and scattered voids, consistent with the partial removal of the organic (collagen) phase. At 400 °C (Figure 1c), the surface shows further increased granularity and surface roughness relative to DKB300, with more small voids and exposed grain boundaries, consistent with the large increase in surface area measured by BET between 300 and 400 °C (Section 2.4). At 500 °C (Figure 1d), the surface shows the most pronounced granularity of the series, with densely packed mineral grains, sharper particle boundaries, and visible voids and cracks between grains, consistent with near-complete removal of the organic matrix and the progressive exposure of the underlying hydroxyapatite framework. Across the series, the SEM images show a shift from a fibrous, collagen-dominated surface in raw bone to an increasingly granular, mineral-dominated surface as pyrolysis temperature increases, consistent with the carbon, nitrogen, and sulfur retained in DKB (Section 2.5). This progressive pore exposure is confirmed quantitatively in the following sections by N_2_ physisorption, BET data (Section 2.4), and EDS Ca/P ratios, which show mesoporosity developing steadily with pyrolysis temperature rather than collapsing at 400 °C.

### 2.2. FTIR Analysis of Duck Bone Biochar and XRD

**Figure 2:**
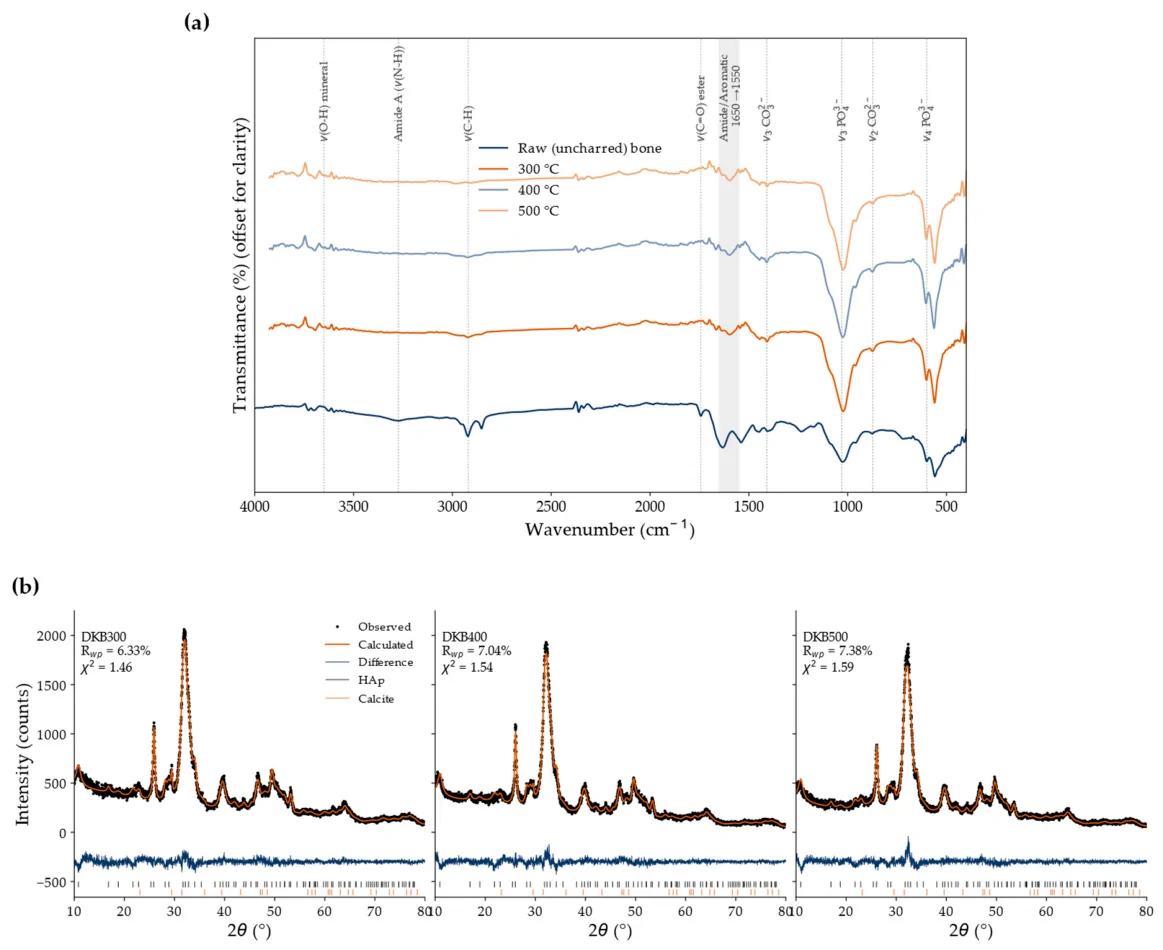
(**a**) FTIR spectra of raw duck bone and DKB and (**b**) Rietveld refinement fits of the XRD patterns of DKB produced at 300, 400, and 500 °C (dots, observed; line, calculated; lower trace, difference; tick marks, hydroxyapatite (black) and calcite (orange) reflections; refined values in Table 1).

FTIR and XRD (Figure 2a,b) show how pyrolysis transforms the organic and mineral fractions of duck bone. Raw bone shows the collagen/lipid bands Amide A (3273 cm^−1^), ν(C–H) (2920 cm^−1^), ester ν(C = O) (1743 cm^−1^), and Amide I/II (1650/1550 cm^−1^). After pyrolysis these bands weaken markedly, and Amide I/II merge into a weak band at 1591–1594 cm^−1^. This band is attributed to aromatic C = C/N-heterocyclic structures of carbonized protein rather than intact amide. This agrees with the progressive removal of the collagen coating seen by SEM (Figure 1, Section 2.1). It also agrees with CHNS, which shows C (17.38 = 12.95 wt%), N (2.64 = 1.74 wt%), and S (0.10–0.22 wt%) retained as a condensed N-containing carbon phase. The mineral bands, ν_3_/ν_4_ PO_4_^3−^ (~1030 and ~600/560 cm^−1^) and CO_3_^2−^ (1409/873 cm^−1^), remain unchanged, indicating carbonated hydroxyapatite.

XRD (Figure 2b) complements the FTIR results. The patterns show a broad amorphous contribution from the carbonized organic fraction and a sharp reflection at 26.0°, together with reflections at 29.0–29.6° (210/121), 32.0–32.3° (211/112/300, the strongest reflection), 39.7–39.8° (310), 46.7–46.8° (222), 49.5–49.7° (213), and 53.2–53.5° (004) that index to hydroxyapatite (HAp) and Ca_10_(PO_4_)_6_(OH)_2_ (space group P6_3_/m; reference structure of Hughes, Cameron and Crowley [24]). Full-pattern Rietveld refinement of the three patterns (10–80° 2θ; Figure 2b and Table 1) converged with Rwp = 6.3–7.4% and χ^2^ = 1.46–1.59. The model comprised HAp, a polynomial background plus a broad Gaussian term for the amorphous carbon contribution, pseudo-Voigt profiles with anisotropic crystallite-size broadening, and a March–Dollase preferred-orientation correction along [001] (r = 1.10–1.14). The sharp reflection at 26.0° is assigned to HAp (002), and not to graphite (002); the texture-corrected HAp model reproduces its intensity, and adding graphite (P6_3_/mmc) gave no meaningful improvement of the fit, so no crystalline graphite is detected and the carbon is present as an amorphous phase, consistent with the FTIR evidence above. Free lime (CaO) and portlandite (Ca(OH)_2_) are likewise not supported; no CaO (200) reflection at 37.4° is present, and Ca(OH)_2_ refined to a scale factor of zero within error. A small additional reflection at 29.4° is accounted for by minor calcite (CaCO_3_, R-3c), which amounts to 1.8 ± 0.2, 0.9 ± 0.2, and 1.1 ± 0.2 wt% of the crystalline phases at 300, 400, and 500 °C and lowers Rwp from 6.8–7.5% to 6.3–7.4%. HAp is therefore the dominant crystalline phase (98.2–99.1 wt%), consistent with the Ca/P ratios obtained by EDS (Section 2.5). The refined HAp lattice parameters are a = 9.448–9.481 Å and c = 6.869–6.887 Å (Table 1). Apparent crystallite sizes (Scherrer equation, K = 0.9, λ = 1.5406 Å, not corrected for instrumental broadening) are 7.4–7.6 nm perpendicular to c and 22.8–25.2 nm along c, i.e., elongated along the c-axis, and change little with pyrolysis temperature. This agrees with the FTIR PO_4_^3−^ and CO_3_^2−^ bands discussed above, which together indicate that pyrolysis leaves a carbonated hydroxyapatite mineral scaffold intact while progressively removing and condensing the organic (collagen) fraction. Uptake is therefore governed, as before, by the exposure of phosphate, hydroxyl, carbonate, and Ca^2+^ exchange sites on this mineral scaffold, rather than by surface area alone (Section 2.6 and Section 2.7).

**Figure 3:**
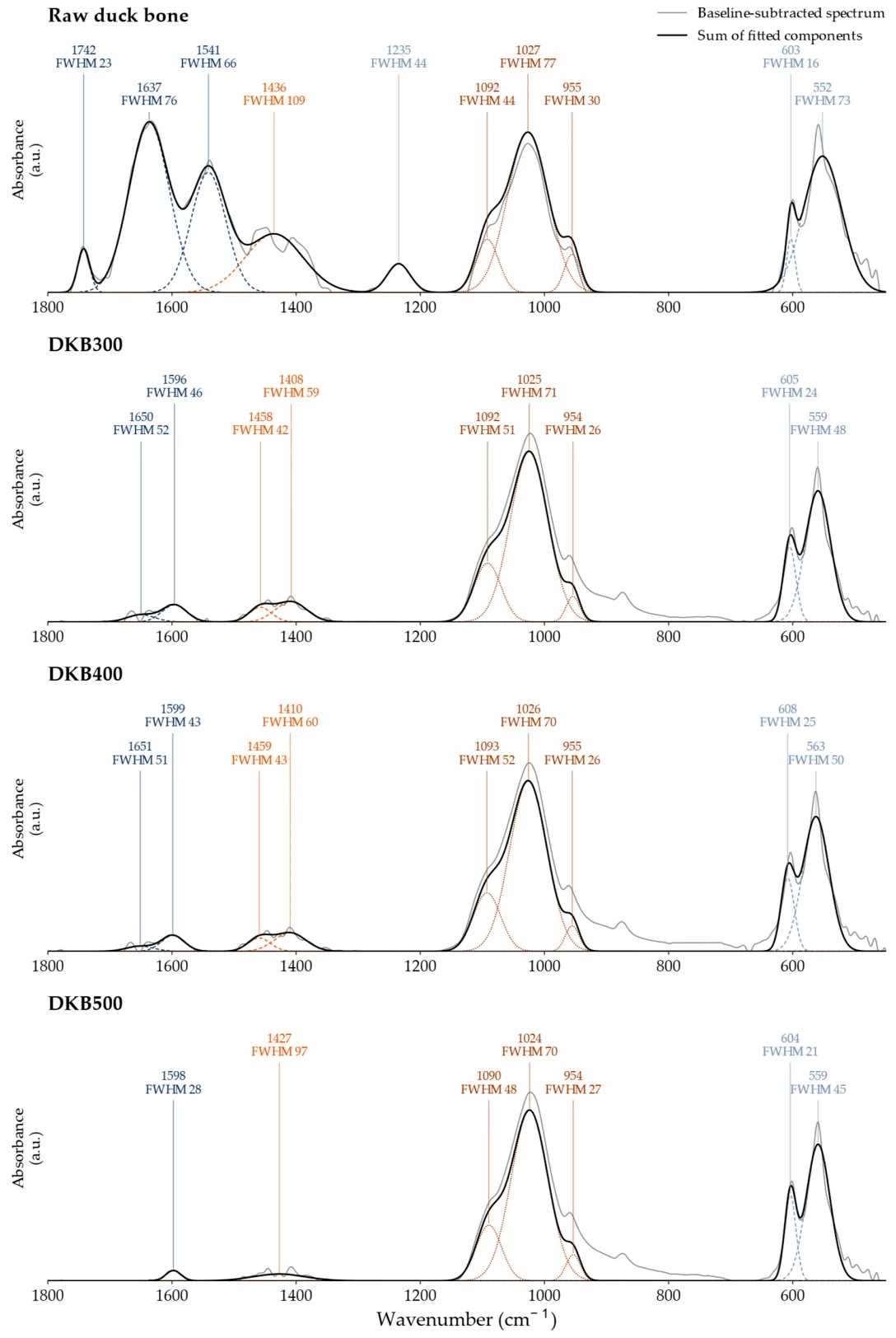
Gaussian deconvolution of the baseline-subtracted FTIR fingerprint region (450–1800 cm^−1^) for raw duck bone, DKB300, DKB400, and DKB500. Dashed lines: amide/aromatic and ν_3_ CO_3_^2−^ components. Light navy dashed: ν_4_ PO_4_^3−^ components. Dark orange dotted: ν_1_/ν_3_ PO_4_^3−^ envelope components. Black: sum of fitted components.

Because the fingerprint region contains few clearly resolved bands, Gaussian deconvolution (local linear baseline; nonlinear least-squares) was applied to the baseline-subtracted 450–1800 cm^−1^ region of each spectrum (raw duck bone, DKB300, DKB400, DKB500) to quantify band position and FWHM across pyrolysis temperature (Figure 3). A single-peak fit of the broad ν_3_ PO_4_^3−^ envelope (≈1030 cm^−1^) is poor (R^2^ = 0.959–0.964; FWHM ≈ 97–107 cm^−1^), but three Gaussians resolve it into physically assignable components (R^2^ = 0.998–0.999): a weak ν_1_ PO_4_^3−^ at ≈954–955 cm^−1^, the dominant ν_3_ PO_4_^3−^ at ≈1024–1027 cm^−1^, and a higher-frequency ν_3_ shoulder at ≈1090–1092 cm^−1^ consistent with the known triple splitting of the ν_3_ phosphate mode in hydroxyapatite. The summed ν_1_ + ν_3_ area increases with pyrolysis temperature (5.5, 11.1, 12.0, and 12.3 a.u. for raw, DKB300, DKB400, and DKB500), tracking the rising mineral fraction as the organic matrix is lost (CHNS, Section 2.5). The ν_4_ PO_4_^3−^ region likewise resolves into a doublet (≈552–563 and ≈603–608 cm^−1^) that sharpens with temperature, consistent with the well-ordered hydroxyapatite lattice identified by XRD (Section 2.2). In the carbonate/amide region, raw bone shows a single, broad ν_3_ CO_3_^2−^ band near 1436 cm^−1^ with amide I (≈1637 cm^−1^) and amide II (≈1541 cm^−1^); after pyrolysis at 300 and 400 °C this carbonate band splits into two components (≈1408 and ≈1458 cm^−1^), reflecting inequivalent carbonate environments in carbonated hydroxyapatite, while the collapsed amide/aromatic region separates into two weak components (≈1596–1599 and ≈1650–1651 cm^−1^). At 500 °C these bands approach the noise floor (absorbance < ~0.01), so a single, broad, unresolved carbonate feature is reported for DKB500, rather than an over-interpreted, noise-limited two-component fit.

### 2.3. Nitrogen Adsorption–Desorption Isotherms

**Figure 4:**
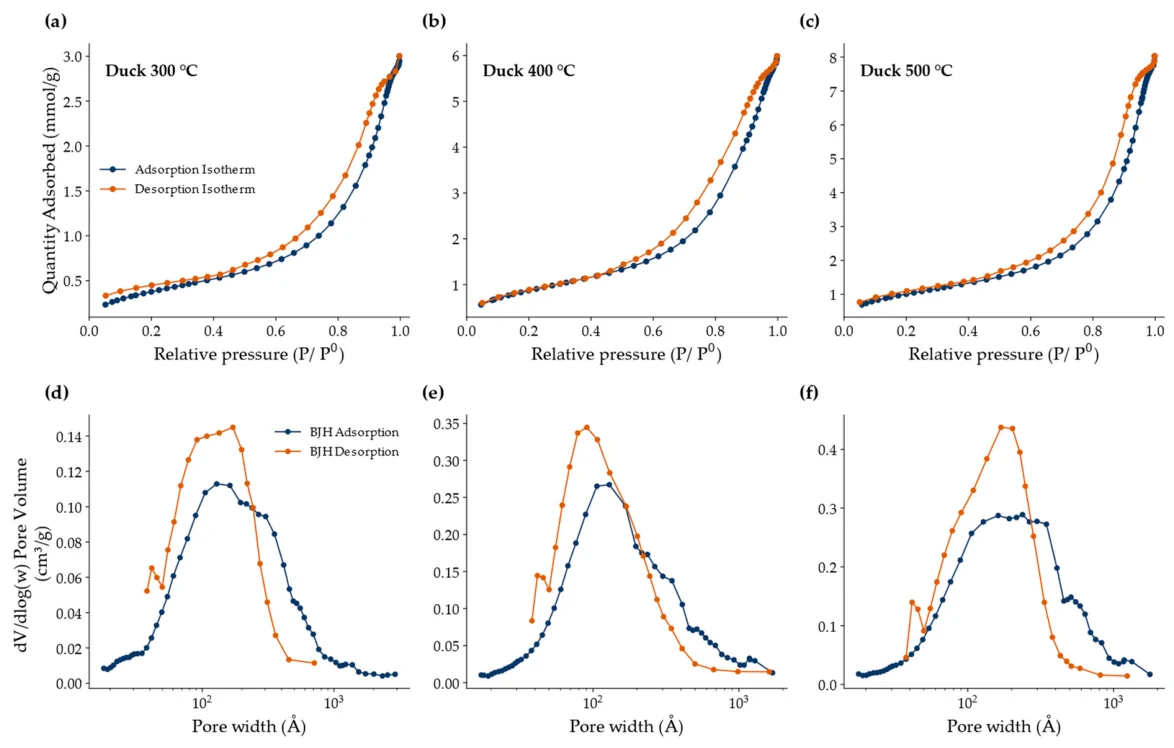
Nitrogen adsorption–desorption isotherms and BJH pore width distributions of duck bone char (DKB) produced at different pyrolysis temperatures. (**a**–**c**) Adsorption–desorption isotherms for DKB300, DKB400, and DKB500. (**d**–**f**) Corresponding BJH adsorption and desorption pore width distributions (dV/dlog(w) vs. pore width) for DKB300, DKB400, and DKB500.

Figure 4 shows the N_2_ adsorption–desorption isotherms of DKB at 300, 400, and 500 °C. All three isotherms follow the IUPAC Type IV(a) pattern of a mesoporous material; uptake rises gradually and near-linearly at low relative pressure (P/P_0_ < 0.15), with no distinct initial step or plateau indicative of micropore filling, followed by multilayer adsorption and capillary condensation with a hysteresis loop as P/P_0_ → 1.0. This absence of a micropore-filling step is corroborated in Section 2.4 by both a t-plot (Harkins–Jura) micropore volume of essentially zero and a Dubinin–Astakhov analysis that finds no resolvable micropore population at any pyrolysis temperature; porosity in DKB is therefore essentially mesoporous, not hierarchical micro-mesoporous. At 300 °C (Figure 4a), adsorption is the lowest of the three samples (maximum uptake about 3.0 mmol/g) and the hysteresis loop is narrow, indicating limited mesoporosity. At 400 °C (Figure 4b), both the adsorbed quantity (maximum about 6.0 mmol/g) and the hysteresis loop widen relative to 300 °C, with the desorption branch remaining above the adsorption branch across the full pressure range, consistent with a well-developed, well-connected mesopore network. At 500 °C (Figure 4c), porosity increases further, giving the widest hysteresis loop of the three samples and the maximum adsorption (about 8.0 mmol/g). This shows that mesoporosity in DKB develops progressively with increasing pyrolysis temperature, consistent with the SEM results in Section 2.1. Thus, the isotherms confirm the SEM results; porosity development in DKB increases steadily from 300 to 500 °C, with no evidence of pore collapse at 400 °C.

### 2.4. Specific Surface Area, Pore Size, and Total Pore Volume of Biochar

The BET data for DKB (Table 2 and Figure 4) quantitatively confirm the trends seen by SEM (Section 2.1) and N_2_ isotherm shape (Section 2.3). Surface area increases progressively with pyrolysis temperature, from 32.972 to 73.468 to 84.181 m^2^ g^−1^ at 300, 400, and 500 °C. Pore volume follows the same trend, rising from 0.101 to 0.203 to 0.269 cm^3^ g^−1^. Average pore diameter narrows slightly from 12.271 to 11.046 nm between 300 and 400 °C and then widens to 12.789 nm at 500 °C. Thus, porosity in DKB develops steadily with pyrolysis temperature, with no genuine collapse at 400 °C, and DKB shows its greatest pore development at 500 °C, consistent with the N_2_ isotherm shapes in Section 2.3. The BJH pore width distributions (Figure 4d–f) show a single, unimodal mesopore population for all three samples, peaking at about 130, 110, and 180 Å for DKB300, DKB400, and DKB500, respectively, with the desorption branch distribution consistently narrower and shifted to slightly larger apparent widths than the adsorption branch distribution, as expected for the positive H3-type hysteresis seen in Figure 4a–c. This is corroborated by the t-plot (Harkins–Jura) analysis of the same isotherms, which gives micropore volumes of −0.0057, −0.0077, and −0.0079 cm^3^ g^−1^ for DKB300, DKB400, and DKB500—statistically indistinguishable from zero—with external surface areas (42.944, 86.418, and 97.366 m^2^ g^−1^) exceeding the corresponding total BET surface areas, the standard signature of a non-microporous material. We additionally fitted the Dubinin–Astakhov (DA) equation to the low-relative-pressure region (P/P_0_ ≤ 0.3) of both branches to test for a resolvable micropore population. The fits converge (R^2^ > 0.999) but return characteristic energies of only E_0_ ≈ 1.0–2.4 kJ mol^−1^ with order parameters *n* < 1 for all three samples, far below the E_0_ ≈ 8–30 kJ mol^−1^ and *n* ≈ 1.5–3 expected for genuine micropore volume filling; the fitted E_0_ values are also too low for the Stoeckli pore width correlation to be applied. This indicates that the low-pressure curvature of the DKB isotherms reflects ordinary monolayer–multilayer coverage rather than micropore condensation, so DA analysis does not resolve a distinct micropore population in DKB at any pyrolysis temperature. Pore development in DKB is therefore essentially mesoporous in character, consistent with the BJH distributions and the 11–13 nm average pore diameters in Table 2.

### 2.5. CHNS Elemental Analysis and EDS Elemental Composition of Duck Bone Char

Elemental composition, as presented in Table 3, offers additional perspectives on the organic-to-mineral transformation process, which explains the changes observed in the textural properties. In DKB, carbon content decreases with increasing pyrolysis temperature from 17.384 to 12.953 wt%. The same trend is observed in the case of hydrogen content, which decreases from 2.382 to 1.287 wt%. These decreases in carbon and hydrogen content can be explained by the fact of the continuing dehydrogenation and volatilization of organic residues with increasing pyrolysis intensity, which is confirmed by the persistence but weakening of the CH_2_ stretching vibration in FTIR spectra (Section 2.2). Also, nitrogen content gradually decreases with temperature increase, from 2.640 to 1.740 wt%, which confirms the downshift and weakening of the Amide II band observed by FTIR, as the nitrogen in bone char comes only from the remains of collagen and proteins. Sulfur was detected in DKB at all temperatures, in the range of 0.101–0.217 wt%.

**Table 3:** Elemental composition (CHNS analyzer) of duck bone char produced at different pyrolysis temperatures.

Sample	Carbon (wt%)	Hydrogen (wt%)	Nitrogen (wt%)	Sulfur (wt%)
DKB (300 °C)	17.384	2.382	2.64	0.177
DKB (400 °C)	16.507	2.332	2.489	0.217
DKB (500 °C)	12.953	1.287	1.74	0.101

**Figure 5:**
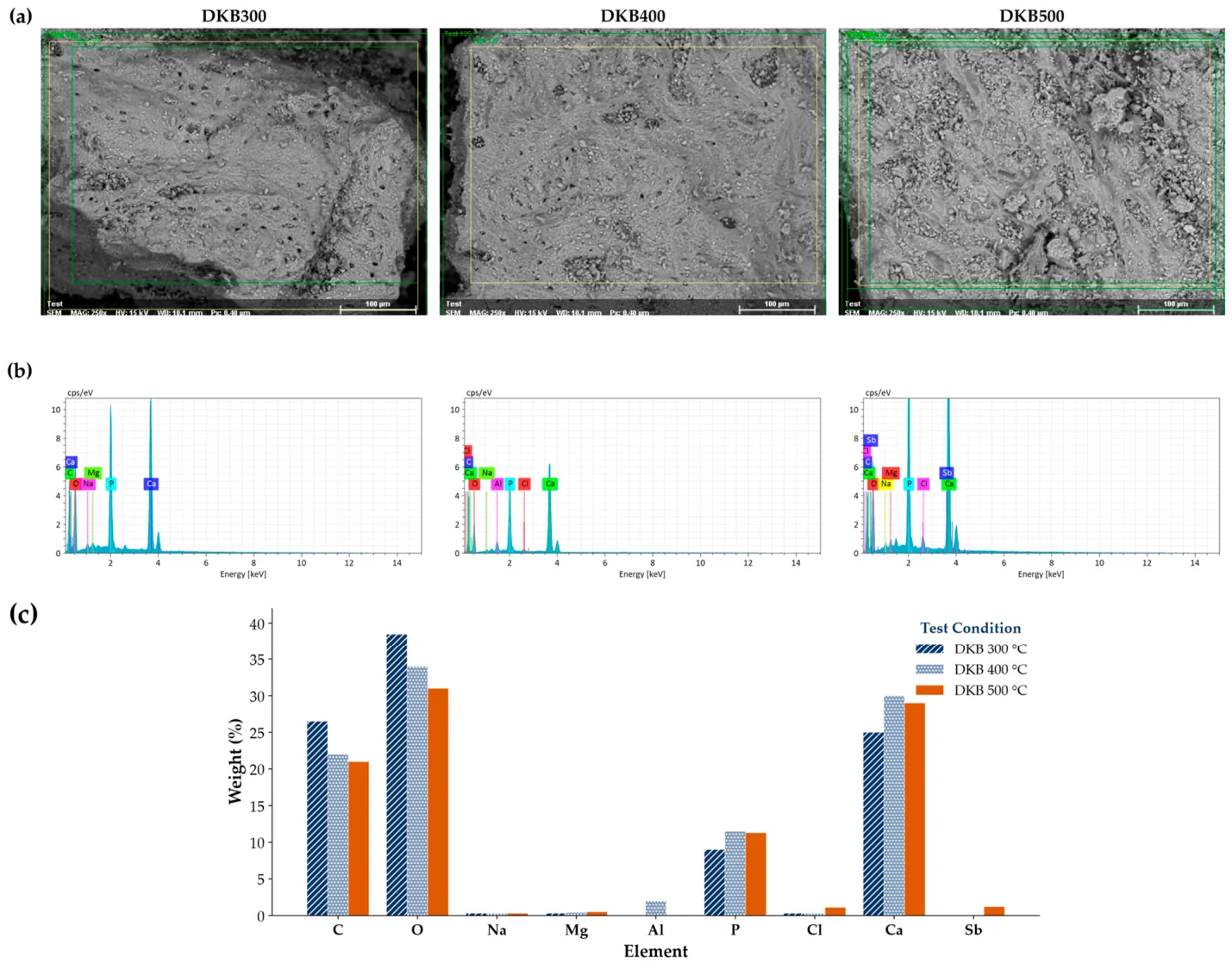
Area scan EDS of DKB produced at 300, 400, and 500 °C. (**a**) SEM (backscattered electron) images at 250× with the analyzed regions outlined (about 0.46 × 0.31 mm); (**b**) corresponding EDS spectra with the automatic peak labels of the acquisition software; (**c**) elemental composition (wt%) obtained from these area scans. Elemental maps were not acquired.

The EDS analysis of DKB at the three pyrolysis temperatures is shown in Figure 5. The spectra were acquired as area scans over the rectangular regions marked on the SEM images in Figure 5a (about 0.46 × 0.31 mm at 250×), so the values in Figure 5 are averages over these areas rather than single-spot analyses; elemental maps were not acquired. Carbon and oxygen are the major elements, together with calcium and phosphorus from the hydroxyapatite phase, which is typical of mineralized bone-derived char. Carbon decreases continuously from 26.6 to 21.8 to 20.8 wt% at 300, 400, and 500 °C, and oxygen from 38.5 to 33.8 to 31.0 wt%, reflecting the volatilization of organic matter and corroborating the CHNS carbon trend. Calcium rises from 25.0 to 30.0 wt% at 400 °C and then dips slightly to 29.1 wt% at 500 °C, while phosphorus rises from 9.1 to 11.6 and then to 11.3 wt%. Together, the loss of C and O and the gain in Ca and P reflect the continuous decomposition of the organic matrix and enrichment of the mineral phase by weight. The corresponding molar Ca/P ratios are 2.12, 2.00, and 1.99 at 300, 400, and 500 °C, above the stoichiometric value of hydroxyapatite (1.67), consistent with carbonate substitution and the semi-quantitative nature of EDS. Antimony was also detected (0.0 wt% at 300–400 °C and about 1.2 wt% at 500 °C), which is atypical for bone-derived material. Because EDS is semi-quantitative and susceptible to surface heterogeneity, this may be a measurement artifact; the Sb L lines (3.60–3.84 keV) overlap the Ca Kα and Kβ lines (3.69 and 4.01 keV), so the Sb label in the automatic peak identification of the DKB500 spectrum (Figure 5b) is most likely a deconvolution artifact rather than evidence of antimony. It should be confirmed on additional areas; if reproducible, its origin needs to be identified. Trace elements (Na, Mg, Al, Cl; below 2 wt%) are attributed to residual minerals and matrix salts in the bone tissue.

### 2.6. Adsorption Performance of Duck Bone Char

**Figure 6:**
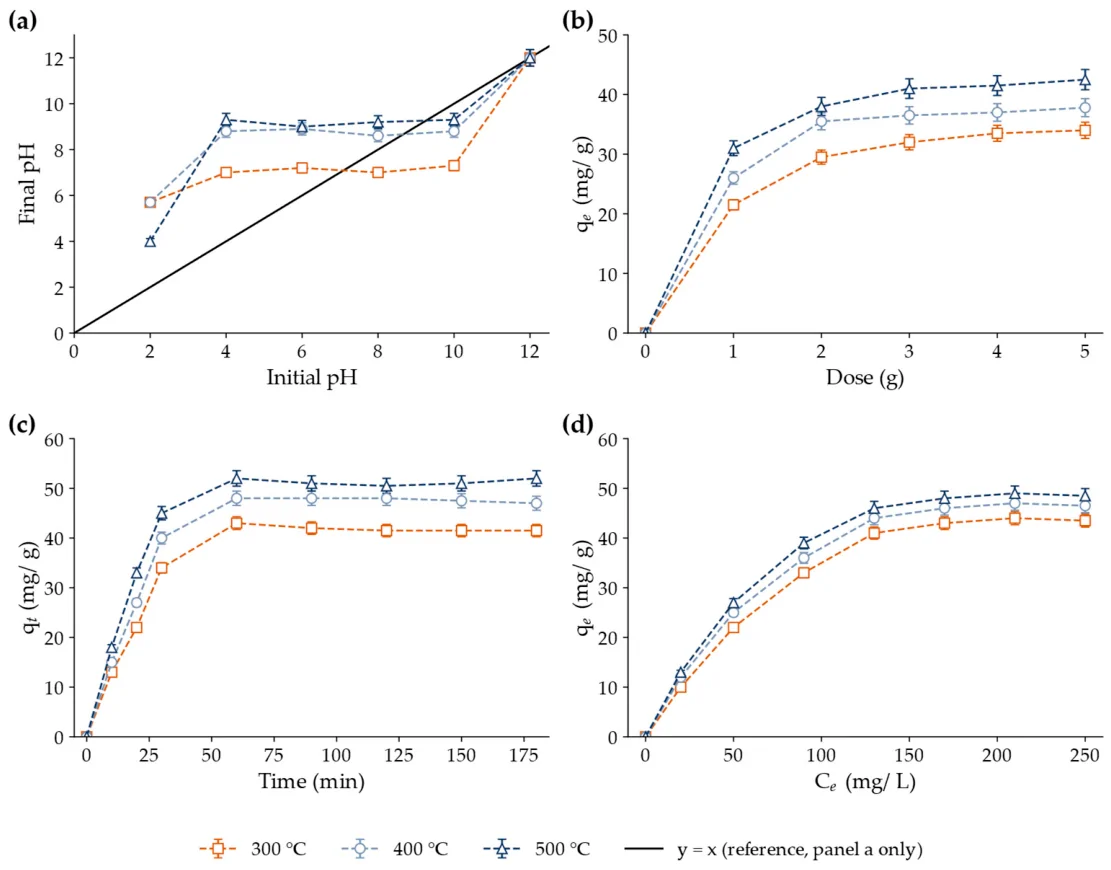
Adsorption performance of duck bone char (DKB) for Cu(II) removal under different experimental conditions: (**a**) effect of initial solution pH, (**b**) effect of adsorbent dosage, (**c**) adsorption kinetics, and (**d**) adsorption isotherms.

The adsorption performance of DKB in relation to various pyrolysis temperatures in regard to the effects of pH, adsorbent dosage, contact time and equilibrium concentration on Cu(II) adsorption is represented in Figure 6. From Figure 6a, it is seen that the points of zero charge (pH_PZC_) are approximately 7.1, 8.8, and 9.5 for DKB300, DKB400, and DKB500, respectively, due to significant changes in the surface chemistry because of the pyrolysis process. In this way, below the pH_PZC_, the protonation of oxygen functional groups created positive surface charges that compete with Cu(II) for adsorption sites, while higher pH reduces this competition and the electrostatic repulsion of Cu^2+^, and thus increases the adsorption efficiency at near-neutral pH. However, no further pH was used because of the potential precipitation of Cu(OH)_2_. From dosage experiments in Figure 6b, it is clear that the removal of Cu(II) increases rapidly up to about 2 g of biochar, since more adsorption sites become available together with larger surface area; after that, adsorption becomes steady after around 3 g. The biochar pyrolyzed at 500 °C had the highest adsorption capacity, mainly because removal of the organic matrix exposed more apatite sites; its larger pore volume chiefly accelerated uptake (Section 2.7). The kinetic studies in Figure 6c show a very rapid adsorption process of Cu(II) in the first 30–40 min and then a gradual approach to equilibrium within about 60 min, and thus indicate a dual-stage behavior of fast adsorption at first at abundant external sites, and later slow diffusion inside the pores, characteristic of heterogeneous porous adsorbents. Equilibrium within about 60 min shows good kinetics for practical application in water treatment systems. From isotherm studies in Figure 6d, the adsorption capacity increased gradually with the increment of Cu(II) concentration up to 150–200 mg L^−1^, and then reached saturation. Implying the progressive approach toward an adsorption plateau indicates the saturation of the available adsorption sites; however, subsequent nonlinear isotherm analysis demonstrates substantial energetic heterogeneity rather than an ideal homogeneous Langmuir surface. Thus, the better performance of DKB500 is related mainly to the greater exposure of apatite sites, while its better pore development, higher surface roughness, and easier access to adsorption sites mainly increase the uptake rate. BET (highest pore volume at 500 °C) and SEM (progressive pore development) support faster access to these sites, but capacity does not scale with surface area; the area-normalized uptake falls from 1.28 to 0.65 and 0.61 mg m^−2^ from DKB300 to DKB500 (Table 2; Section 2.7). Increasing pyrolysis temperature therefore improves Cu(II) adsorption mainly via surface complexation and ion exchange with calcium-bearing mineral phases, with intraparticle diffusion in the developed pores controlling the rate.

### 2.7. Kinetic and Isotherm Adsorption Study

Six nonlinear kinetic models (PFO, PSO, Avrami, Weibull with lag time, DEM, and BWS) were fitted to the Cu(II) uptake data (R^2^, adjusted R^2^, RMSE, χ^2^; Table 4, Figure 6). PSO fits poorly (R^2^ = 0.951–0.966; RMSE = 3.27–3.60) and overestimates q_e_ by 15–19% (50.4–58.6 mg g^−1^), so a single integer-order rate law cannot describe the process. BWS, Weibull, and Avrami fit almost equally well (R^2^ = 0.9976–0.9989; RMSE = 0.68–1.03; χ^2^ = 0.094–0.235); DEM ranks slightly lower (R^2^ = 0.9951–0.9968). All three stretched-exponential exponents exceed 1 (nₐᵥ = 1.48–1.25, m = 1.58–1.30, α = 1.44–1.21), indicating sigmoidal, multistep uptake rather than a single elementary step, consistent with rapid initial uptake (30–40 min) followed by a slower approach to equilibrium at ~60 min (Figure 6c). The negative Weibull lag time (t_0_ < 0) at all temperatures rules out a real induction period; the sigmoidal shape instead reflects the sequential occupation of heterogeneous sites. Exponents decrease with temperature as the organic overlayer is removed and more apatite surface is exposed (Section 2.2), bringing uptake closer to first-order. The rate data agree; kₐᵥ is essentially unchanged from 300 to 400 °C (0.042 min^−1^) and rises to 0.049 min^−1^ at 500 °C, while the BWS characteristic time falls from 24.6 to 21.0 min, consistent with the larger mesopore volume of DKB500 (0.269 cm^3^ g^−1^; Table 2) easing Cu(II) transport to internal sites.

**Figure 7:**
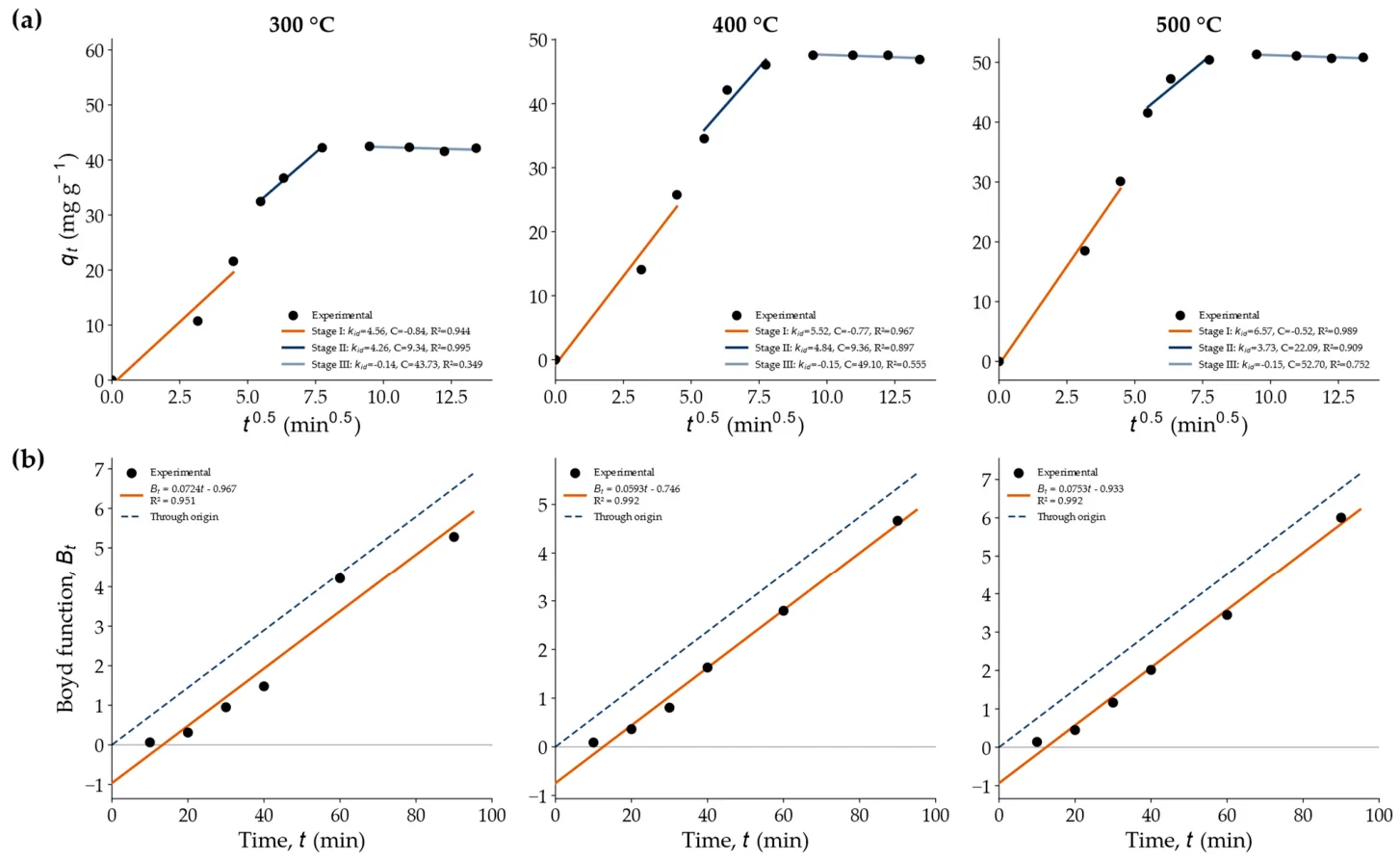
Diffusion model: (**a**) Weber–Morris intraparticle diffusion model and (**b**) Boyd film-diffusion model for Cu(II) adsorbed onto DKB.

The Weber–Morris intraparticle diffusion model (q_t_ = k_id_·t^0.5^ + C; Figure 7a) was fitted piecewise over three time regions (0–20, 30–60, and 90–180 min), since a single line across the full range fits poorly (R^2^ = 0.79–0.80). At all three temperatures, stage 1 (external/rapid surface uptake) gives k_id_ = 4.56–6.57 mg g^−1^ min^−0^·^5^ with a near-zero intercept (C = −0.84 to −0.52 mg g^−1^; R^2^ = 0.94–0.99); stage 2 (intraparticle diffusion) gives a lower k_id = 3.73–4.84 mg g^−1^ min^−0^·^5^ but a markedly positive intercept (C = 9.3–22.1 mg g^−1^; R^2^ = 0.90–0.995); stage 3 (90–180 min) is an equilibrium plateau (k_id_ ≈ 0, C = 43.7–52.7 mg g^−1^). Because C is substantially positive in the diffusion-controlled second stage at every temperature, the plots do not pass through the origin, showing that intraparticle diffusion is not the sole rate-controlling step. This is corroborated by the Boyd film-diffusion model (Reichenberg linearization of Bt vs. t; Figure 7b), fitted over 10–90 min using the DEM-fitted equilibrium capacity (q_e_ = 42.6, 47.8, and 51.4 mg g^−1^) to define F = q_t_/q_e_; the Bt–t plots are linear (R^2^ = 0.95–0.99) but do not pass through the origin, with negative intercepts (−0.97, −0.75, and −0.93 at 300, 400, and 500 °C), indicating that film (external) diffusion contributes to the rate. Because Bt becomes very sensitive to F as F approaches 1, the last two points of the fitted window (F ≥ 0.96) carry large uncertainty, and an effective diffusivity was not calculated because the particle radius was not determined. Together, the two analyses indicate that Cu(II) uptake onto DKB is governed by a combination of external film and intraparticle diffusion rather than either process alone, consistent with the sigmoidal kinetics above (nₐᵥ > 1).

**Figure 8:**
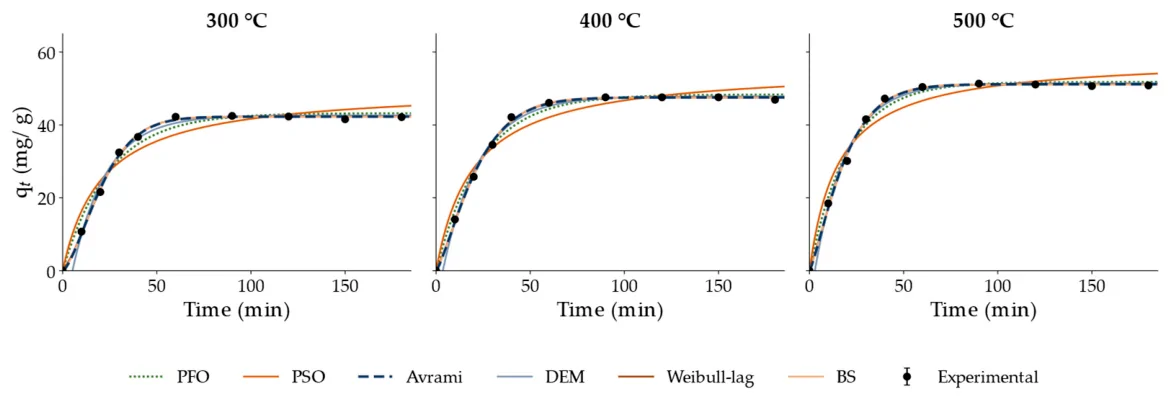
Experimental and nonlinear model-predicted kinetic profiles for Cu(II) adsorption onto DKB produced at 500 °C.

Equilibrium capacity increases from 42.3 to 47.5 to 51.2 mg g^−1^ but does not scale with surface area. DKB300, despite the lowest surface area and pore volume of the series (32.972 m^2^ g^−1^, 0.101 cm^3^ g^−1^), still reaches 42.3 mg g^−1^, giving the highest area-normalized uptake (1.28 mg m^−2^ vs. 0.65 and 0.61 mg m^−2^ for DKB400 and DKB500). Capacity is therefore governed mainly by the availability of apatite phosphate, hydroxyl, and Ca^2+^ exchange sites, while the larger pore volume developed at a higher pyrolysis temperature chiefly governs uptake rate (Section 2.6) and accounts for the modest additional capacity gained at 400 and 500 °C. DKB500 is thus the fastest and highest-capacity adsorbent in the series (Figure 8).

**Figure 9:**
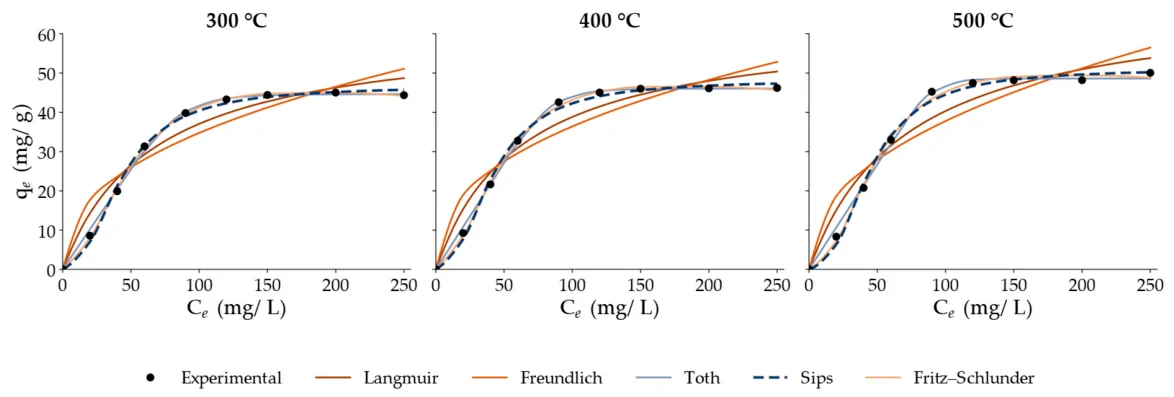
Experimental equilibrium data and nonlinear adsorption–isotherm model predictions for Cu(II) adsorption onto DKB produced at 500 °C.

Five nonlinear isotherm models (Freundlich, Langmuir, Toth, Sips, Fritz–Schlunder) were fitted to the equilibrium data at 300, 400, and 500 °C (Table 5, Figure 9). The two-parameter models fit poorly: Freundlich gives R^2^ = 0.891–0.898, RMSE = 5.87–6.78; Langmuir gives R^2^ = 0.949–0.957 and overestimates capacity (q_m_ = 61.6–69.2 mg g^−1^) because the data saturate more sharply than a Langmuir curve. Among the three-parameter models, Toth fits best (R^2^ = 0.9966–0.9987; RMSE = 0.73–1.30), ahead of Sips (R^2^ = 0.9951–0.9967). The four-parameter Fritz–Schlunder model gives the highest R^2^ and lowest SSE/χ^2^ at all temperatures (R^2^ = 0.9969–0.9994; RMSE = 0.52–1.34; χ^2^ = 0.076–0.342), with its advantage over Toth largest at 300 °C (SSE 1.35 vs. 4.93) and marginal at 500 °C (9.04 vs. 10.11, where Toth has a slightly higher adjusted R^2^ and lower RMSE). All heterogeneity exponents exceed unity (Toth n_th_ = 5.77, 6.72, 9.90; Sips n_s_ = 2.24–2.41; Fritz–Schlunder a = 1.70–1.97), describing an isotherm that rises slowly at low C_e_ and then saturates abruptly, typical of cooperative uptake such as Ca^2+^ – Cu^2+^ ion exchange accompanied by the surface nucleation of Cu–phosphate phases, and consistent with the sigmoidal kinetics above (Avrami n_av_ > 1). Exponents increase with temperature as the organic overlayer is removed and more apatite surface is exposed (Section 2.2), explaining why the extra flexibility of the four-parameter model helps most at 300 °C, where the partly covered surface gives the least regular uptake. The Toth capacity rises from 44.65 to 46.05 to 48.60 mg g^−1^, and the Sips capacity from 46.61 to 48.13 to 50.99 mg g^−1^, closely matching the kinetic equilibrium capacities (42.3–51.2 mg g^−1^). As in the kinetic analysis, capacity does not scale with surface area: a 2.6-fold increase in BET surface area (33.0 to 84.2 m^2^ g^−1^; Table 2) raises the Toth capacity by only about 9% (44.65 to 48.60 mg g^−1^), so maximum capacity is governed mainly by the accessibility of apatite phosphate, hydroxyl, and Ca^2+^ exchange sites, not by pore development alone.

**Figure 10:**
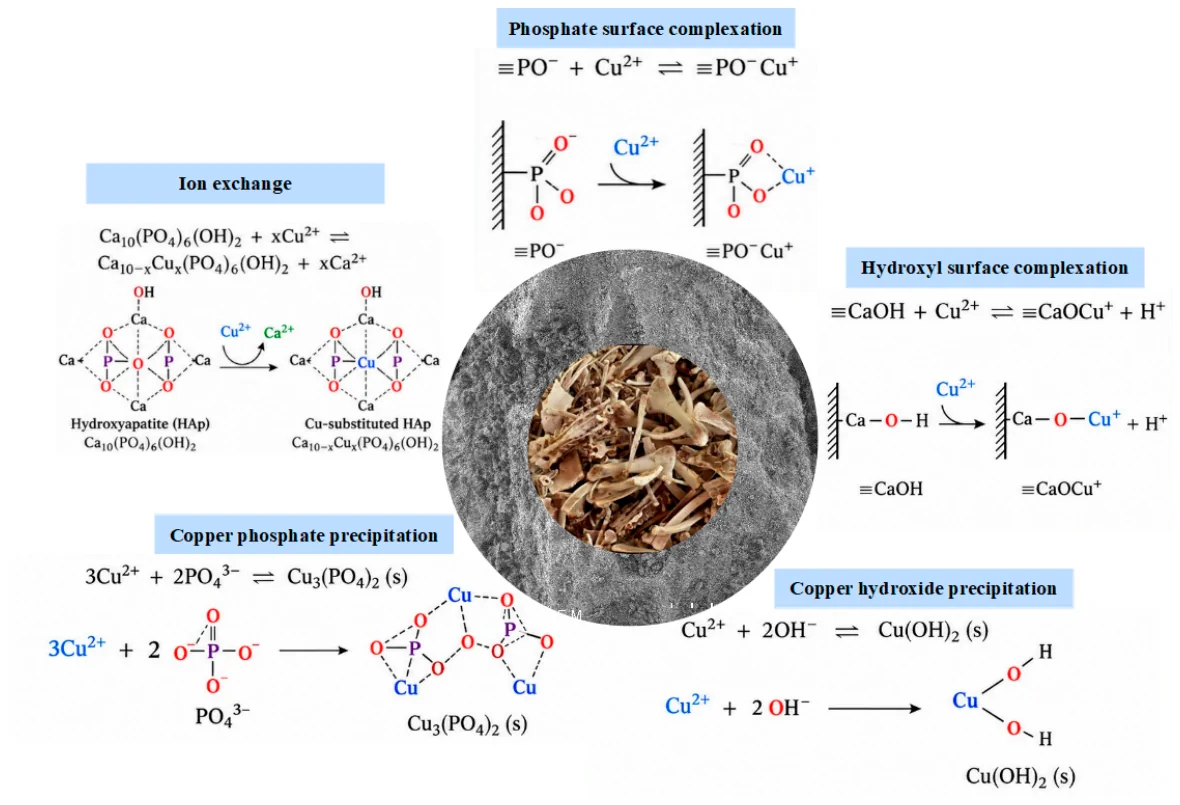
Proposed Cu(II) removal pathways on duck bone char (DKB). Ion exchange and phosphate/hydroxyl surface complexation are the proposed main pathways; Cu_3_(PO_4_)_2_ nucleation is a proposed minor pathway, and Cu(OH)_2_ precipitation is shown for completeness but is not expected at the experimental pH (Section 2.10).

Cu(II) adsorption onto DKB is best explained by several chemically controlled pathways on the carbonated hydroxyapatite scaffold, with physical processes playing only a secondary role (Figure 10). Ion exchange between Cu^2+^ and lattice/surface Ca^2+^ [Ca_10_(PO_4_)_6_(OH)_2_ + xCu^2+^ → Ca_10−x_Cu_x_(PO_4_)_6_(OH)_2_ + xCa^2+^], by analogy with Ca^2+^-for-M^2+^ exchange reported for other divalent metals on hydroxyapatite-type sorbents (e.g., [36]), is consistent with the high Ca and P contents measured by EDS and the positive ΔS° (0.30–0.36 kJ mol^−1^ K^−1^), reflecting the release of Ca^2+^ and hydration water, though this is indirect evidence, since aqueous Ca^2+^ and the exchange ratio itself were not measured. Cu^2+^ is also proposed to form inner-sphere complexes with deprotonated phosphate, hydroxyl, and carbonate groups (≡P–OH + Cu^2+^ → ≡P–O–Cu^+^ + H^+^), whose bands persist in FTIR at all pyrolysis temperatures, comparable to EXAFS-reported Cu^2+^ surface complexation on synthetic hydroxyapatite [36]. The partial dissolution of apatite/carbonate can raise the local interfacial pH (pH_p_zc ≈ 7–9.5), favoring the surface nucleation of Cu_3_(PO_4_)_2_-type copper-phosphate clusters (bulk Cu(OH)_2_ precipitation is not expected under the sub-saturation conditions used; Section 2.10)—supported by the sigmoidal kinetics (n_av_ = 1.25–1.48), sharply saturating isotherms (n_th_ = 5.8–9.9), and strongly endothermic ΔH° (71–90 kJ mol^−1^), too high for physisorption alone. Electrostatic attraction is unfavorable, since the surface is net positive below pH_p_zc, and pore filling/van der Waals forces are minor, since the mesopores (average diameters 11.0–12.8 nm; Table 2) are much wider than hydrated Cu^2+^ and act mainly as diffusion channels, explaining DKB500′s faster uptake (0.269 cm^3^ g^−1^) rather than higher capacity. This site-controlled mechanism explains why capacity follows apatite-site accessibility rather than surface area, and why HCl regeneration recovers only part of the performance, since the acid desorbs Cu^2+^ but also dissolves part of the apatite scaffold. Because q_e_ was obtained from filtrate mass balance alone, with no Ca^2+^ quantification or post-uptake XPS/XRD on the Cu-loaded sorbent, these pathways are proposed and literature-supported rather than directly demonstrated, and their relative contributions should be confirmed by future post-uptake XPS/XRD/FTIR, Ca^2+^-release measurements, and precipitation controls.

### 2.8. Thermodynamic Analysis of Cu(II) Adsorption onto Duck Bone Char

**Figure 11:**
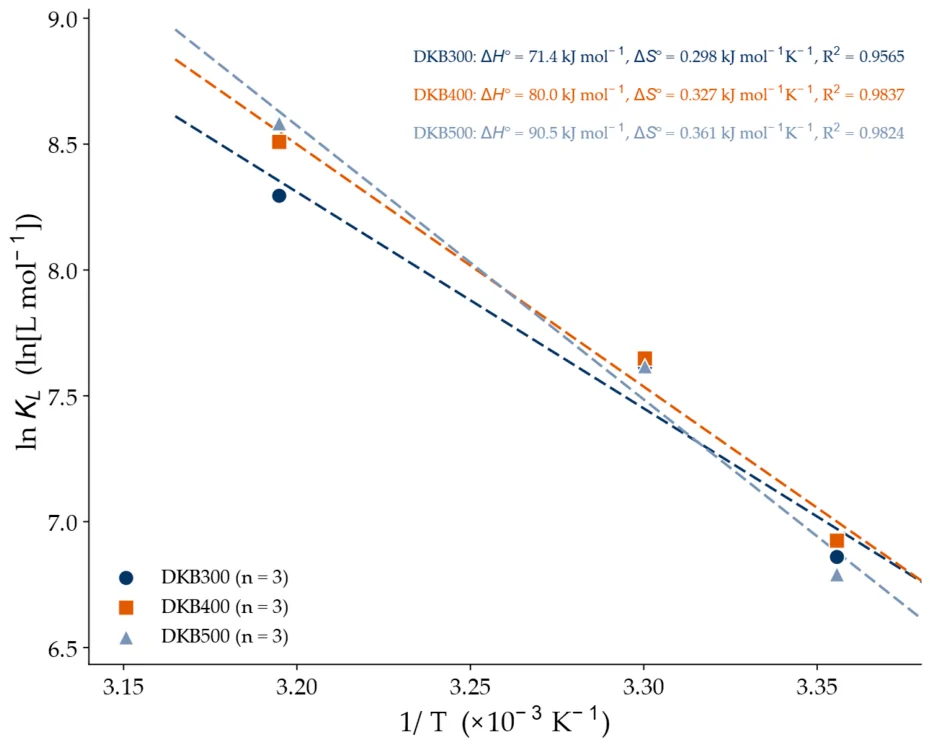
Van’t Hoff plots (ln K_L_ vs. 1/T) for Cu(II) adsorption onto duck bone char (DKB300, DKB400, DKB500).

The Langmuir constants (K_L_) and thermodynamic parameters of Cu(II) adsorption onto DKB at 298, 303, and 313 K are given in Table 6. K_L_ increases with solution temperature for all three chars, and ΔG° is negative in every case and becomes more negative from 298 to 313 K, indicating a spontaneous, favorable overall removal process. With pyrolysis temperature, ΔG° at 313 K becomes more negative (−21.59, −22.14, and −22.33 kJ mol^−1^ for DKB300, DKB400, and DKB500), while differences at 298 and 303 K are small. ΔH° is positive (71.45 to 90.45 kJ mol^−1^ from 300 to 500 °C), indicating an endothermic process. ΔS° is also positive (0.30–0.36 kJ mol^−1^ K^−1^) and increases with pyrolysis temperature, indicating greater interfacial disorder, likely from water release during Cu(II) uptake. The high ΔH° is consistent with Cu(II) binding mainly through surface chemistry (e.g., chemisorption on phosphates/hydroxides) rather than physical porosity, in line with the moderate surface area of DKB (33–84 m^2^ g^−1^; Section 2.4). However, because q_e_ is calculated from filtrate mass balance without confirming that adsorption is the only removal pathway, these parameters describe the temperature dependence of the overall apparent removal process and are not independent evidence of chemisorption. Firmer attribution awaits post-uptake characterization and precipitation controls, which were not performed in this study. The uncertainties in ΔH°, ΔS°, and ΔG° reported in Table 6, together with the uncertainty budget for the underlying experimental methods and solution preparation, are detailed in Section 3.4.6 . The underlying Van’t Hoff plots (ln K_L_ vs. 1/T) from which ΔH° and ΔS° were obtained by linear regression for each pyrolysis temperature are provided in Figure 11.

### 2.9. Desorption, Regeneration, and Cycle-Domain Modeling

**Figure 12:**
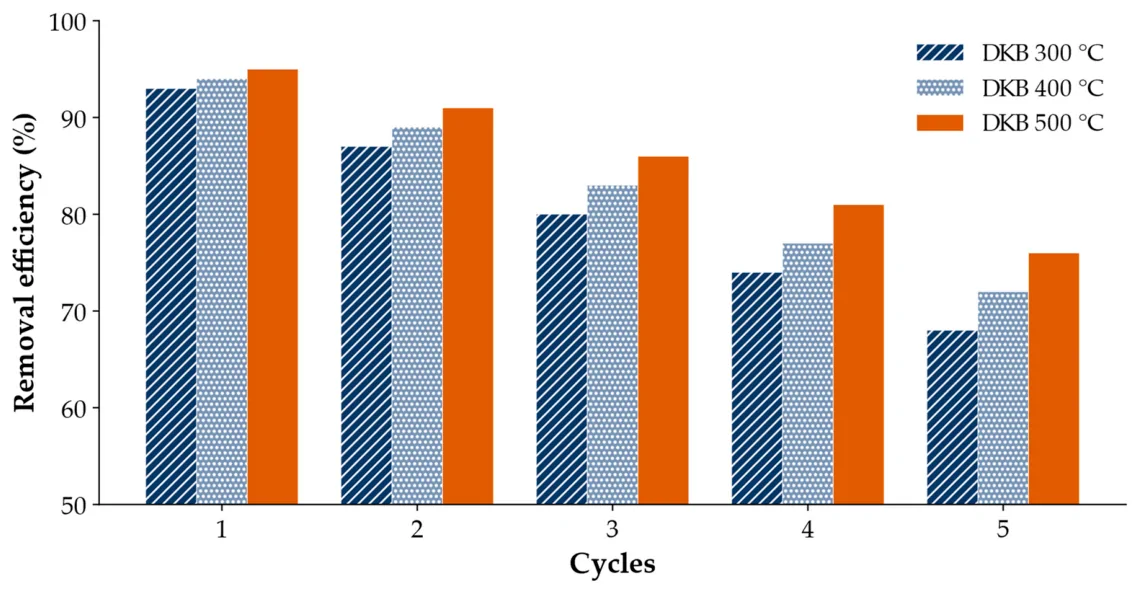
Cu(II) removal efficiency and regeneration performance of DKB produced at 300, 400, and 500 °C over five consecutive adsorption–desorption cycles.

Figure 12 shows the Cu(II) removal efficiency of DKB300, DKB400, and DKB500 over five adsorption–desorption cycles with 0.1 M HCl regeneration. Efficiency declines almost linearly with cycle number, with no plateau within five cycles. The average loss per cycle is about 6.3, 5.5, and 4.8 percentage points for DKB300, DKB400, and DKB500, respectively. From cycle 1 to cycle 5, efficiency falls from 93% to 68%, 94% to 72%, and 95% to 76%, so the three chars retain 73.1%, 76.6%, and 80.0% of their initial performance. Stability increases steadily with pyrolysis temperature, in parallel with surface area and pore volume, so these textural changes cannot be separated from the accompanying exposure of the mineral surface on the basis of the present data. Crystallite size is also nearly constant across 300–500 °C (Section 2.2), so the difference is unlikely to come from better mineral ordering. Two factors are more likely. First, removing the labile organic overlayer at higher temperature leaves a more acid-resistant, mineral-rich surface. Second, the large mesopore volume of DKB500 (0.269 cm^3^ g^−1^) allows the acid to reach the Cu(II) held inside the particle and desorb it more completely. The gradual loss of efficiency is consistent with the proposed mechanism (Section 2.7). Cu^2+^ that has been incorporated into the apatite lattice by ion exchange, or precipitated as Cu-phosphate clusters, is only partly removed by HCl. The acid can also dissolve part of the hydroxyapatite scaffold, and both effects gradually reduce the number of active sites. Separating these two effects, however, requires measuring Cu desorption efficiency and Ca/P leaching in each cycle, which was not done here.

Four empirical cycle-domain models (Table 7) were fitted to the desorption/regeneration data (E = normalized removal efficiency vs. cycle number, *n*): a first-order decay (M1), a power-law decay (M2), an Avrami-type stretched exponential (M3), and a residual-activity model with an asymptotic floor (M4).

The first-order decay model (M1) described the efficiency loss closely for all three DKB chars (R^2^ = 0.9948–0.9983). The Avrami-type model (M3) gave marginally higher R^2^ (0.9976–0.9992), but its shape exponent was close to unity (m = 1.07–1.12), so the decay is essentially first-order and M3 adds little beyond M1. The power-law model (M2) fitted least well (R^2^ = 0.91–0.93). In the residual-activity model (M4), the fitted floor converged to zero for all samples (E_i_ = 0), so M4 reduced to a first-order decay with no identifiable residual activity within five cycles, consistent with the absence of a plateau in Figure 12; it is therefore not interpreted further. The M1 decay constant was k = 0.056–0.078 cycle^−1^ and decreased with pyrolysis temperature (0.0783, 0.0674, and 0.0558 cycle^−1^ at 300, 400, and 500 °C). The 500 °C char therefore loses efficiency most slowly, consistent with its greater stability (Section 2.9) and highest cycle-5 efficiency (Figure 12). DKB chars contain a high fraction of chemisorbed Cu(II) (Section 2.8) and are thus less reversible under acid regeneration. The fitted E_0_ (≈1.01) is the extrapolated efficiency of the fresh adsorbent (*n* = 0), and the predicted cycle-1 efficiencies (93.5–95.6%) closely match the measured values (93–95%), confirming the fit. Overall, k is a single, physically meaningful measure of regeneration stability: a smaller k (e.g., DKB500) indicates a more durable adsorbent, and a larger k (e.g., DKB300) indicates faster loss of active sites during repeated HCl regeneration.

### 2.10. Thermodynamic Phase Diagram of Cu(II)

**Figure 13:**
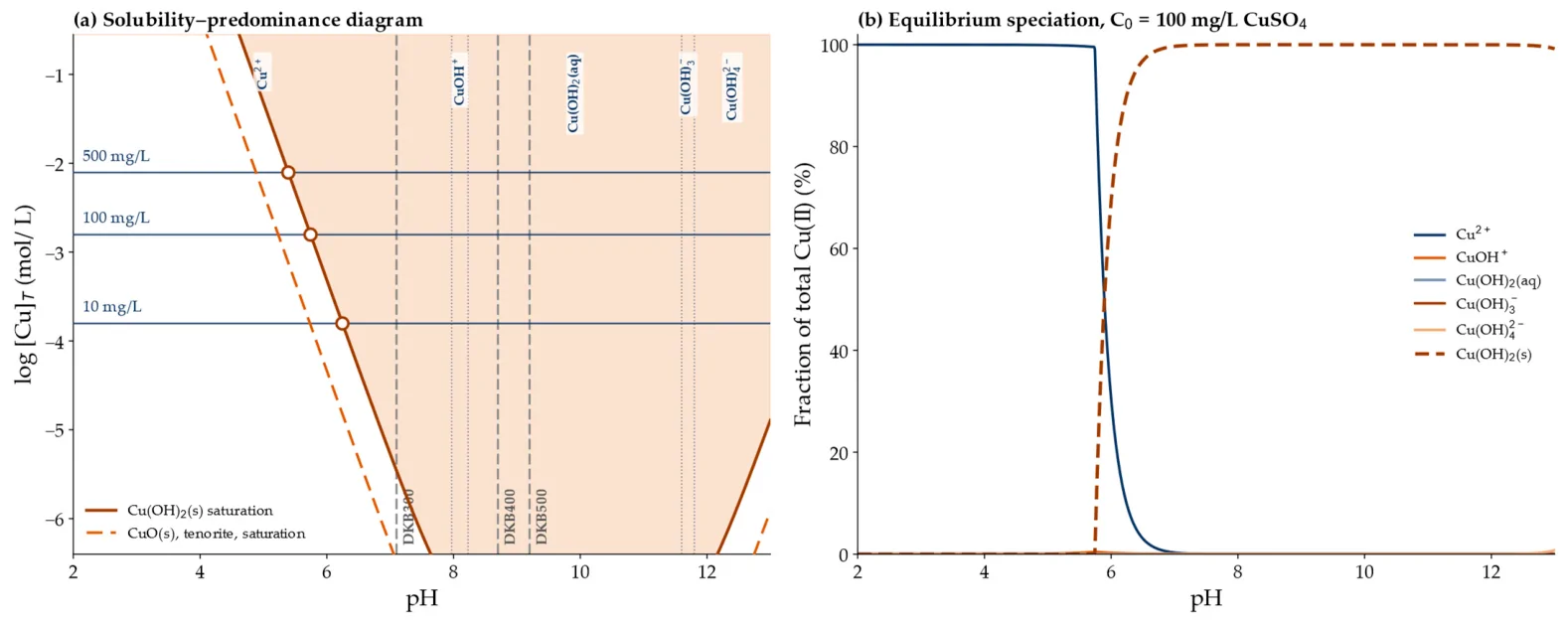
Thermodynamic phase diagrams of Cu(II) at 25 °C: (**a**) solubility-predominance diagram with Cu(OH)_2_(s)/CuO(s) saturation boundaries, predominance fields, C_0_ = 10/100/500 mg L^−1^ lines, and DKB300/400/500 buffering pH; (**b**) equilibrium Cu(II) speciation at C_0_ = 100 mg L^−1^ CuSO_4_.

To complement the empirical pH dependence described in Section 2.6 and the Van’t Hoff-derived thermodynamic parameters in Section 2.8, the aqueous-phase thermodynamics of Cu(II) were examined independently of the biochar, using the stepwise hydrolysis constants of Powell et al. [37] (log β1–β4 = –7.97, –16.2, –27.8, and –39.6 at I = 0, 25 °C) together with solubility products from the literature for Cu(OH)_2_(s) and CuO(s) (tenorite). This places the Cu(II)–water system used experimentally (C_0_ = 10–500 mg L^−1^; Section 3.3) within its solubility and predominance fields, and so separates a genuine adsorption contribution from solution-phase precipitation. Figure 13a shows the resulting solubility-predominance diagram. Both the Cu(OH)_2_(s) boundary (solid line) and the less soluble CuO(s) boundary (dashed line) rise steeply with pH, so that for a fixed total Cu(II) concentration the system is undersaturated at low pH and crosses into the supersaturated, solid-forming regime once a characteristic saturation pH is exceeded. For the three concentrations used experimentally (10, 100, and 500 mg L^−1^; Section 3.3), the calculated saturation pH values are 6.25, 5.74, and 5.39, respectively (open circles, Figure 13a): the more concentrated solutions precipitate at a lower pH because less additional hydroxide is required to exceed Ksp. These values are markedly lower than the pH_PZC_ of DKB300, DKB400, and DKB500 (7.1, 8.8, and 9.5, respectively; Section 2.6, Figure 6a; gray dashed lines in Figure 13a), so that for every char and every concentration tested, the pH at which the biochar surface becomes favorably charged for Cu^2+^ uptake lies inside the thermodynamically supersaturated region. This gives a quantitative basis for, and is consistent with, the experimental decision not to extend the pH-effect tests beyond near-neutral conditions because of the risk of Cu(OH)_2_ precipitation (Section 2.6).

**Figure 14:**
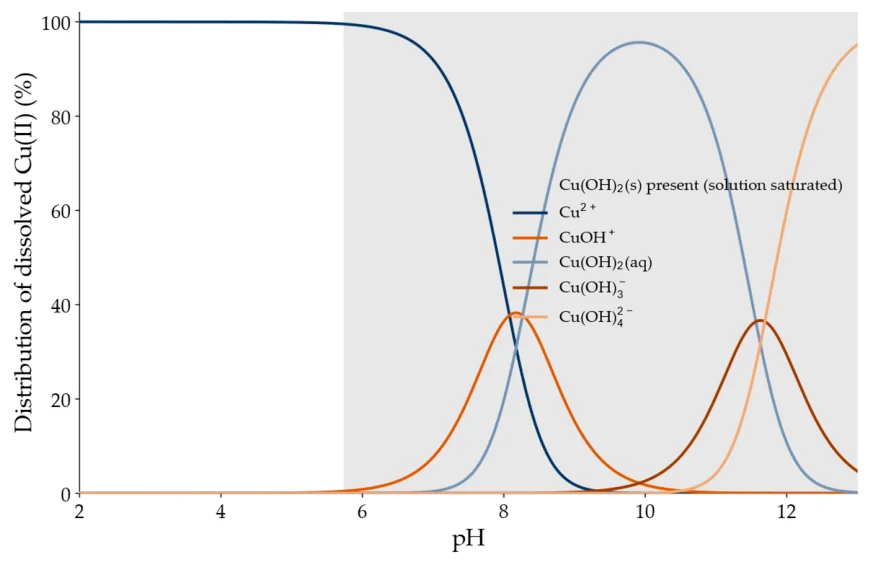
Distribution of dissolved Cu(II) species vs. pH at C_0_ = 100 mg L^−1^ CuSO_4_ (Davies correction); shaded region = Cu(OH)_2_(s) saturated.

Figure 13b and Figure 14 resolve the same system into its individual dissolved species at C_0_ = 100 mg L^−1^ Cu(II) as CuSO_4_, treated as a closed system with the Davies equation used to correct the hydrolysis and solubility constants for the ionic strength contributed by the sulfate background electrolyte (I ≈ 6.3 × 10^−3^ M; activity coefficients γ_1_ = 0.919, γ_2_ = 0.715). Below pH ≈ 5.7, which is the saturation pH for this concentration (Figure 13a), free Cu^2+^ accounts for essentially all of the Cu(II) present, both as a fraction of C_0_ (Figure 13b) and as a fraction of the dissolved Cu(II) (Figure 14). Once the solution becomes saturated with respect to Cu(OH)_2_(s), the solid fraction rises sharply and dominates the total Cu(II) mass balance above pH ≈ 7 (Figure 13b); within the remaining dissolved fraction (Figure 14), the classical hydrolysis sequence Cu^2+^ → CuOH^+^ → Cu(OH)_2_(aq) → Cu(OH)_3_^−^ → Cu(OH)_4_^2−^ develops with increasing pH, each species peaking in turn near pH 8.2, 9.9, and 11.7, consistent with the mononuclear predominance boundaries calculated at pH 7.97, 8.23, 11.6, and 11.8 (I = 0; Figure 13a). Because the pH-effect experiments in Section 2.6 remained below neutral, free Cu^2+^ is therefore inferred to be the dominant, and for practical purposes the only, Cu(II) species available for uptake throughout this study; the hydrolyzed species in Figure 14 become significant only once the system has already begun to precipitate, and so are not relevant to the kinetic and isotherm data in Section 2.7, which can accordingly be interpreted as describing Cu^2+^ adsorption specifically rather than a pH-dependent mixture of hydrolysis products.

**Figure 15:**
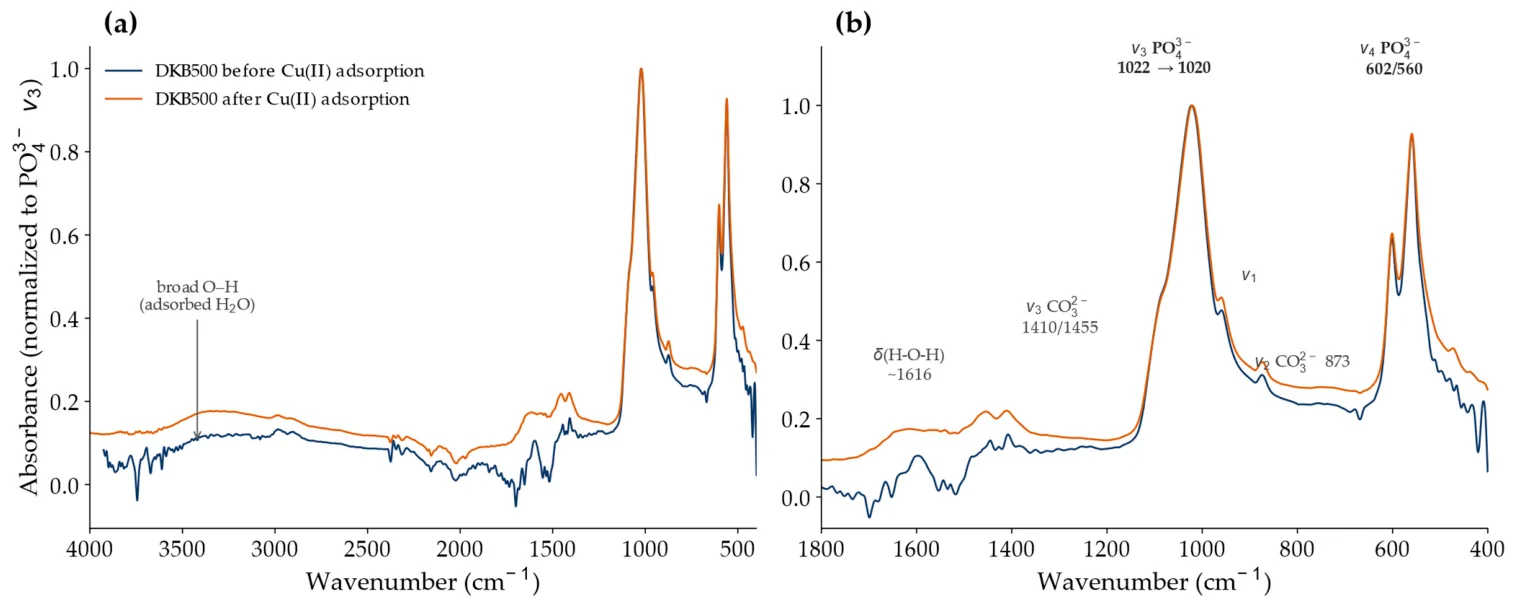
FTIR spectra of DKB500 before/after Cu(II) adsorption (normalized to PO_4_^3−^ ν_3_): (**a**) 4000–400 cm^−1^; (**b**) 1800–400 cm^−1^.

Spectroscopic evidence supports a surface, rather than a bulk-precipitation, origin for Cu(II) uptake under these sub-saturation conditions. Figure 15 compares the FTIR spectrum of DKB500 before and after Cu(II) adsorption, both normalized to the PO_4_^3−^ ν_3_ band. The ν_3_ PO_4_^3−^ envelope shifts only marginally, from 1022 to 1020 cm^−1^, and the characteristic ν_4_ PO_4_^3−^ doublet (602/560 cm^−1^; Section 2.2) is essentially unchanged in position after adsorption. This pattern, a small shift of the asymmetric stretch with an undisturbed bending-mode doublet, is consistent with Cu^2+^ coordinating at existing phosphate/hydroxyl surface sites through ion exchange and surface complexation (Figure 10; Section 2.7), rather than with bulk reconstruction of the apatite framework or formation of a separate Cu(OH)_2_(s) phase on the char, either of which would be expected to perturb the PO_4_^3−^ manifold more substantially. Taken together with the thermodynamic analysis above, the FTIR, isotherm/kinetic (Section 2.7), and thermodynamic parameter (Section 2.8) results converge on a single picture: within the sub-saturation pH window realized experimentally, Cu(II) removal by DKB is governed by ion exchange and surface complexation at calcium-phosphate and hydroxyl sites, and homogeneous Cu(OH)_2_ precipitation becomes thermodynamically possible, and must be guarded against, only once pH approaches 5.4–6.3, depending on the initial Cu(II) concentration (10–500 mg L^−1^).

### 2.11. Significance of the Research

Hydroxyapatite-rich bone chars from chicken, cattle, pig, fish, and duck are well-established Cu(II) adsorbents (Table 8). They remove Cu(II), Pb(II), Cd(II), and Ni(II) mainly through ion exchange, surface complexation, and mineral precipitation. Reported affinities follow the order Cu(II) > Cd(II) > Ni(II) [34], and bone char hydroxyapatite removes Cu(II) and Zn(II) by adsorption and ion exchange [35]. Most earlier studies, however, examined a single bone type at one preparation condition and reported capacity with classical kinetic and isotherm models. The present work adds three elements. First, it evaluates DKB at 300, 400, and 500 °C, and shows that Cu(II) capacity (42.3–51.2 mg g^−1^) follows the accessibility of apatite sites rather than surface area. Second, it links this behavior to a site-controlled, cooperative uptake mechanism. Third, it quantifies regeneration loss with a cycle-domain model. After five HCl cycles, DKB retained 73.1–80.0% of its initial efficiency, comparable to pig bone hydroxyapatite (~74–78%) [36]. Its first-order decay constants (0.056–0.078 cycle^−1^) decreased with pyrolysis temperature, so DKB500 was the most stable. The novelty of this study therefore lies not in Cu(II) removal by duck bone char itself, but in a pyrolysis–structure–adsorption–regeneration framework for selecting and designing reusable bone-derived adsorbents. Within this framework, DKB500 emerges as a low-cost, reusable option.

## 3. Materials and Methods

### 3.1. Preparation of Duck Biochar

**Figure 16:**
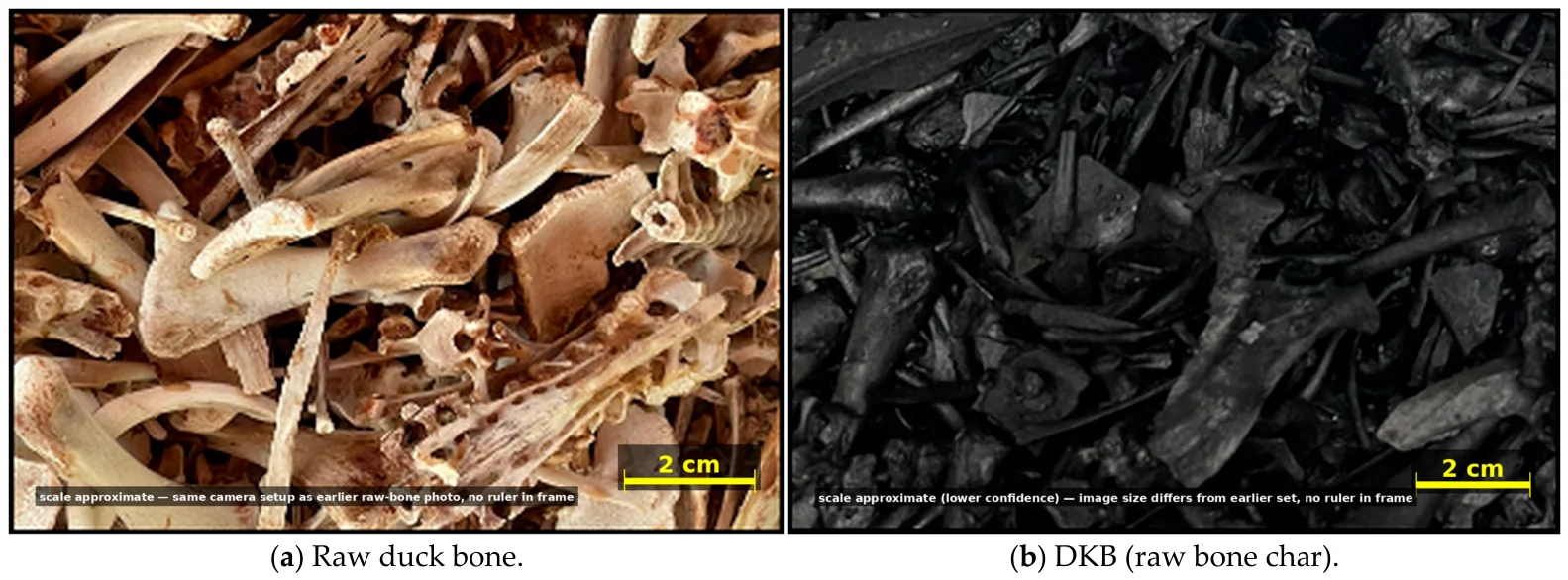
Raw material for DKB biochar production.

**Figure 17:**
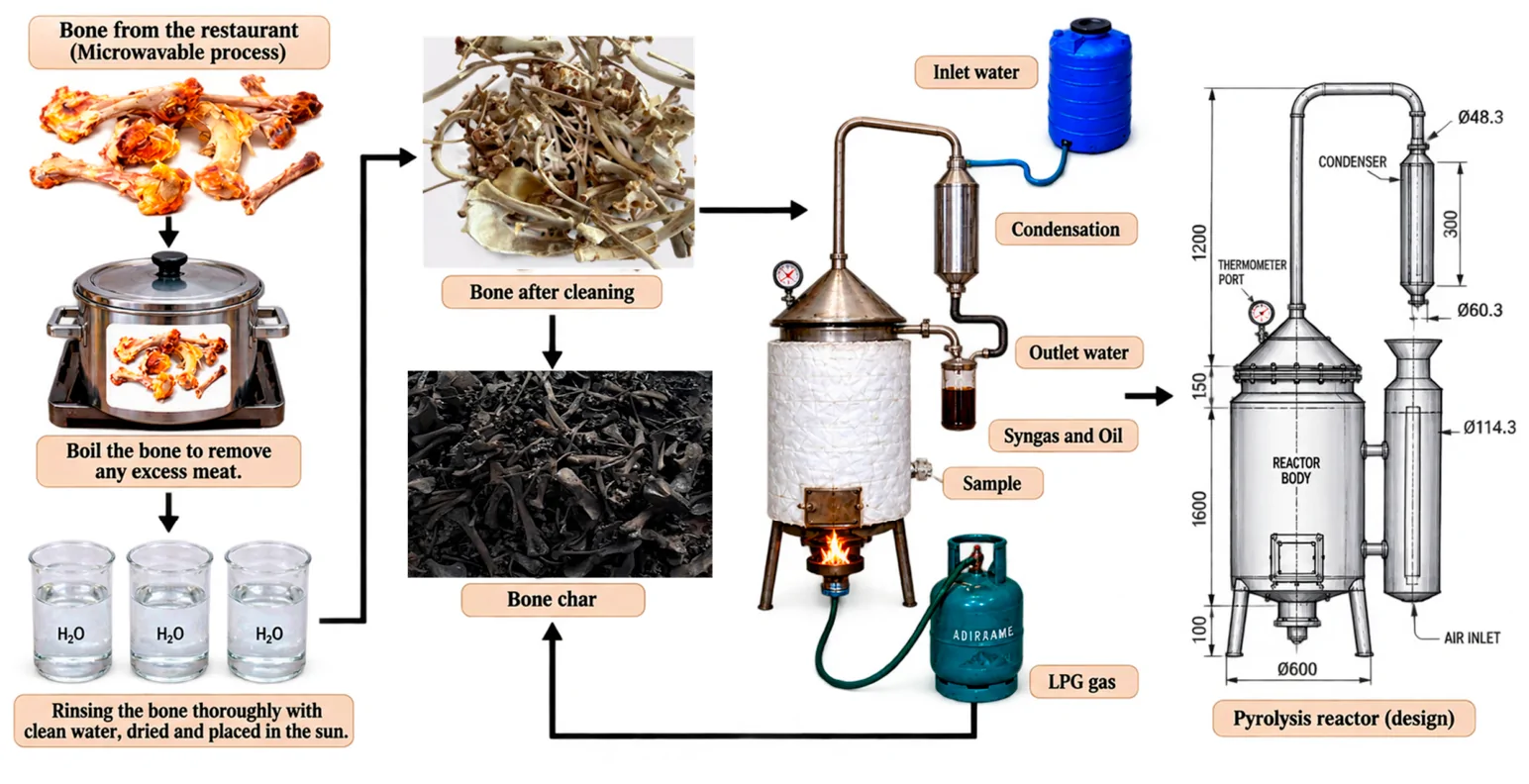
Schematic illustration of the preparation of duck bone char (DKB) by slow pyrolysis (some images generated by ChatGPT Plus (GPT-5, OpenAI, San Francisco, CA, USA; accessed 2026) and Claude Pro (Claude Opus 4, Anthropic, San Francisco, CA, USA; accessed 2026).

Raw duck bones were collected from local restaurants located around Mahasarakham University, Maha Sarakham Province, Thailand. The collected bones, with lengths ranging from approximately 2 to 15 cm (Figure 16), were first manually cleaned to remove adhering meat, fat, and connective tissues. The bones were subsequently washed several times with tap water, followed by rinsing with deionized water to eliminate residual impurities. The cleaned bones were then air-dried under sunlight for 24 h and further dried at room temperature until a constant weight was achieved. Approximately 1.0 kg of dried bones was loaded into a laboratory-scale, modified fixed-bed pyrolysis reactor (internal diameter = 60 cm; height = 100 cm). The reactor was sealed prior to heating, and no external inert gas (e.g., nitrogen or argon) was purged through the system; instead, an oxygen-limited, self-generated (autogenous) atmosphere was achieved as the initial air trapped within the sealed reactor was rapidly consumed and displaced by the volatile pyrolysis gases evolved from the bone matrix upon heating. Slow pyrolysis was performed at three final temperatures (300, 400, and 500 °C). The reactor temperature was increased at a constant heating rate of 12 °C min^−1^ and maintained at the target temperature for 1 h. During pyrolysis, the volatile products generated from thermal decomposition were transferred through a water-cooled condenser to collect condensable liquids, while the non-condensable gases were safely vented. After completion of the reaction, the reactor was allowed to cool naturally to room temperature within the same sealed, oxygen-depleted vessel to prevent re-oxidation of the produced bone char. The resulting duck bone char (DKB) was collected, ground using a marble mortar and pestle, and sieved to obtain a homogeneous powder for subsequent physicochemical characterization and adsorption experiments. The prepared bone char samples were designated as DKB300, DKB400, and DKB500 for duck bone char produced at 300, 400, and 500 °C, respectively. The powdered samples were stored in airtight polyethylene containers in a desiccator until further use. This preparation procedure is illustrated schematically in Figure 17.

### 3.2. Characterization of the Prepared Biochar

The physicochemical properties of the raw duck bone and its corresponding biochars were characterized using Fourier Transform Infrared Spectroscopy (FTIR, Bruker, INVENIOR, Billerica, MA, USA), Scanning Electron Microscopy coupled with Energy Dispersive X-ray Spectroscopy (SEM–EDS, HITACHI TM4000Plus, High-Tech, Tokyo, Japan), Brunauer–Emmett–Teller (BET, TriStar II Plus, Norcross, GA, USA) surface area analysis, and X-ray Diffraction (XRD, Bruker D6 PHASER, Karlsruhe, Germany). FTIR spectra were recorded over the range of 4000–400 cm^−1^ at a resolution of 4 cm^−1^ using 32 scans to identify surface functional groups before and after pyrolysis. Elemental composition (C, H, N, S, Thermo Fisher Scientific, Waltham, MA, USA) was determined by CHNS elemental analyzer as a single determination per sample; typical instrument reproducibility is ±0.1–0.3 wt% (absolute) or ~1–3% (relative), per manufacturer specification. The surface morphology and elemental composition were examined using SEM–EDX (TM4000 backscattered electron imaging at 15 kV and 250×; EDS acquired as area scans over the regions shown in Figure 5a) after sputter-coating the samples with a thin gold layer. The specific surface area, pore volume, and pore size distribution were determined by nitrogen adsorption–desorption measurements at 77 K using the BET and Barrett–Joyner–Halenda (BJH) methods following degassing at 200 °C for 12 h. The crystalline phases and mineralogical composition were analyzed by XRD using Cu Kα radiation (λ = 1.5406 Å) over a 2θ range of 5–80° to evaluate the structural transformations induced by pyrolysis. Full-pattern Rietveld refinement (10–80° 2θ) was performed with a custom Python (version 3.13) implementation using hydroxyapatite (P6_3_/m) and calcite (R-3c) structure models, Kα_1_/Kα_2_ pseudo-Voigt profiles with anisotropic size broadening, a March–Dollase correction along [001], and a Chebyshev background plus a broad Gaussian term for the amorphous carbon contribution; weight fractions were obtained by the Hill–Howard relation and apparent crystallite sizes from the Scherrer equation (K = 0.9) without instrumental broadening correction.

### 3.3. Stock Solution Preparation

A stock solution of Cu(II) at a concentration of 1000 mg L^−1^ Cu(II) was made by dissolving an accurate amount of analytical-grade Cu(II) sulfate pentahydrate (CuSO_4_·5H_2_O, QReC, Auckland, New Zealand) in deionized water. This solution was made in a 1 L volumetric flask and kept in a polyethylene bottle at 4 °C. Desired concentrations (10–500 mg L^−1^) of working Cu(II) solutions were prepared immediately before each adsorption test by the serial dilution of stock solution with deionized water. The pH of the solution was adjusted, if necessary, by 0.1 M HCl (Merck, Darmstadt, Germany) or 0.1 M NaOH (Merck, Darmstadt, Germany). All chemicals used in this work were of analytical reagent (AR) grade and deionized water was used throughout the experiment. To make 1 L of 1000 mg L^−1^ Cu(II) solution from CuSO_4_·5H_2_O (M = 249.68 g mol^−1^), 3.929 g of CuSO_4_·5H_2_O was taken, dissolved in deionized water and diluted to 1 L in a volumetric flask. This solution had a concentration of 1000 mg L^−1^ Cu^2+^ ions and acted as stock solution for all the adsorption studies.

### 3.4. Adsorption Study

The adsorption efficiency of biochar made from duck bones was determined by conducting batch adsorption tests involving aqueous solutions of heavy metals. Studies were done systematically to determine the impacts of contact time, initial metal concentration, pH, dose of adsorbent, and temperature on adsorption efficiency. In every experiment, a specific amount of biochar was added to 100 mL of metal solution in a 250 mL Erlenmeyer flask. The solution was shaken in a thermostatic orbital shaker at 150 rpm and a particular temperature. After adsorption for the specified period of time, filtrate was obtained by filtering the solution through a 0.45 μm membrane filter, and the concentration of metal remaining in the solution was determined using ICP–OES. Adsorption efficiency at time t (q_t_) and equilibrium (q_e_) were determined according to Equations (1) and (2),

(1) $$q_{t}=\frac{\left(C_{0}-C_{t}\right)V}{m}$$

(2) $$q_{e}=\frac{\left(C_{0}-C_{e}\right)V}{m}$$

where C_0_ (mg L^−1^) is the initial metal concentration, C_t_ (mg L^−1^) is the concentration at time t, C_e_ (mg L^−1^) is the equilibrium concentration, V (L) is the solution volume, and m (g) is the mass of biochar.

#### 3.4.1. Adsorption Kinetics

Adsorption kinetics experiments were carried out to investigate the adsorption rate and the kinetic mechanism involved in the uptake of Cu(II) ions using the biochar prepared from duck bones. The batch adsorption experiment was carried out by placing 0.10 g of the biochar in 100 mL of Cu(II) ion solution (100 mg L^−1^) in a 250 mL Erlenmeyer flask. The pH of the solution was first set to the optimum pH using either 0.1 M HCl or 0.1 M NaOH. The suspensions were agitated in a thermostatically controlled orbital shaker at 150 rpm and 298 ± 1 K. Aliquots were collected at predetermined time intervals (10, 20, 30, 40, 60, 90, 120, 150, and 180 min) and filtered through 0.45 μm membrane filters, and the residual Cu(II) concentration was quantified using Inductively Coupled Plasma Optical Emission Spectroscopy (ICP–OES, Optima 8000, Perkin Elmer Inc., Waltham, MA, USA). The adsorption capacity at time t (q_t_, mg g^−1^) was calculated according to Equation (1). The kinetic data were subsequently fitted using the five nonlinear kinetic models in Table 4 to elucidate the adsorption mechanism. The applicability of each kinetic model was evaluated using the Sum of Squared Errors (SSE), coefficient of determination (R^2^), adjusted coefficient of determination (Adj. R^2^), root mean square error (RMSE), and chi-square (χ^2^) statistics.

#### 3.4.2. Adsorption Isotherms

Adsorption equilibrium studies were carried out to determine the adsorption capacity and adsorption behavior of Cu(II) on the prepared biochar. Batch adsorption experiments were performed by contacting 0.10 g of biochar with 100 mL of Cu(II) solutions with initial concentrations ranging from 10 to 500 mg L^−1^ at the optimum solution pH. The mixtures were shaken at 150 rpm and 298 ± 1 K for the equilibrium time established from the kinetic experiments. Following equilibrium, the suspensions were filtered through 0.45 μm membrane filters, and the equilibrium Cu(II) concentration (C_e_) was determined by ICP–OES. The equilibrium adsorption capacity (q_e_, mg g^−1^) was calculated using Equation (2). The equilibrium data were analyzed using five nonlinear isotherm models (Table 5) to investigate the adsorption characteristics, including maximum monolayer adsorption capacity, adsorption intensity, adsorption energy, and surface heterogeneity. Model parameters were estimated by nonlinear regression analysis, and the most suitable adsorption isotherm was selected based on the highest R^2^ and Adj. R^2^ and the lowest RMSE, SSE and χ^2^ values. Nonlinear fitting (kinetic and isotherm models) was performed using Microsoft Excel Solver. Initial parameter guesses were derived from linearized model forms or directly from experimental data (e.g., q_e_ from observed equilibrium capacity, rate constants from initial curve slope), not arbitrary values. Convergence was confirmed by residual-sum-of-squares tolerance (<10^−9^) and by repeating fits from multiple starting values to verify stability; non-converging or physically unrealistic fits were discarded.

#### 3.4.3. Adsorption Thermodynamics

The thermodynamic behavior of the adsorption process was investigated by conducting equilibrium experiments at different temperatures (298, 303, and 313 K) while maintaining identical experimental conditions. The equilibrium constant (Kc, taken as the Langmuir constant at each temperature expressed in L mol^−1^; Table 6) was used to determine the Gibbs free energy change (ΔG°), enthalpy change (ΔH°), and entropy change (ΔS°) according to the Van’t Hoff equations. These thermodynamic parameters were employed to evaluate the spontaneity, heat exchange characteristics, and randomness at the solid–liquid interface during adsorption.

#### 3.4.4. Desorption and Regeneration Study

The reusability of the prepared biochar was determined through several sequential adsorption–desorption cycles. After every adsorption process, the Cu(II)-impregnated biochar was filtered out and rinsed with deionized water. The desorption process was carried out using 0.1 M HCl at a 150 rpm stirring rate for 2 h. The desorbed biochar was then washed with deionized water until reaching neutral pH and dried for 12 h at 105 °C. Finally, it was used again under the same conditions of adsorption. The efficiency of adsorption and desorption was recorded after each cycle in order to assess the regeneration capability and stability of the biochar. At least five sequential adsorption–desorption cycles were performed in order to determine the practical utility of the adsorbent for Cu(II) removal.

#### 3.4.5. Statistical Evaluation of Model Fitting

All nonlinear fits were performed in Microsoft Excel Solver, with initial parameter guesses taken from linearized model forms or directly from the data (e.g., q_e_ from the observed equilibrium uptake; rate constants from the initial slope of the q_t_–t or q_e_–C_e_ curve). Convergence required a residual-sum-of-squares change below 10^−9^ over a maximum of 1000 iterations, with each fit repeated from at least two starting-value sets to confirm parameter stability; fits that failed to converge or returned unrealistic parameters (e.g., negative capacities) were discarded. Fit quality was assessed using SSE = Σ(y_exp − y_calc)^2^, R^2^ = 1 − SSE/SST, adjusted R^2^ = 1 − [(1 − R^2^)(*n* − 1)/(*n* − *p*)], RMSE = √(SSE/(*n* − *p*)), χ^2^ = Σ[(y_exp − y_calc)^2^/y_calc], and the corrected Akaike information criterion AICc = *n*·ln(SSE/*n*) + 2p + [2p(*p* + 1)/(*n* − *p* − 1)], where *n* is the number of data points (10 per kinetic run including t = 0, 9 per isotherm run, 5 per regeneration run) and *p* is the number of fitted parameters (Table 3 and Table 4, and 6). AICc was preferred over standard AIC for its small-sample bias correction, which converges to AIC as *n* increases. For each dataset, the model with the highest R^2^/adjusted R^2^ and lowest RMSE, SSE, χ^2^, and AICc was selected as best; this criterion identifies the most parsimonious fit without independently establishing adsorption mechanism, addressed separately in Section 2.6 and Section 2.8.

#### 3.4.6. Uncertainty Assessment of Experimental Methods and Solution Preparation

Beyond the model-fitting diagnostics in Section 3.4.5, the single-determination nature of the kinetic, isotherm, and thermodynamic measurements (Section 3.4.1, Section 3.4.2 and Section 3.4.3) precludes a replicate-based standard deviation for individual C_e_, q_e_, or K_L_ values. We therefore constructed a bottom-up uncertainty budget, following the general approach of the ISO/IEC Guide to the Expression of Uncertainty in Measurement (GUM), using manufacturer- or standard-specified tolerances rather than values measured in this study (Table 9). The budget covers the analytical balance and Class A glassware used to prepare the 1000 mg L^−1^ Cu(II) stock solution and working dilutions (Section 3.3), the declared purity of the CuSO_4_·5H_2_O reagent, the thermostatic control of the shaking bath (298, 303, and 313 ± 1 K), the pH adjustment step, and the instrumental precision of ICP–OES/AAS and the CHNS analyzer. The combined relative uncertainty from solution preparation alone is ≈1.0% (root-sum-of-squares of the balance, glassware, and reagent-purity terms), dominated by reagent purity; combined with typical ICP–OES/AAS instrumental precision, this gives ≈2.2% on each single-determination C_e_ and, by propagation through Equation (2), on q_e_. This analytical/instrumental uncertainty is distinct from the model-fitting uncertainty reported via RMSE, SSE, χ^2^, and AICc, and from the regression-based uncertainty for ΔH°, ΔS°, and ΔG° in Table 6 (Section 2.8)—the only replicate-type (*n* = 3 temperatures) uncertainty obtainable from the present design. As each kinetic, isotherm, and thermodynamic data point was a single determination rather than a replicate mean, the values in Table 9 remain specification-based estimates rather than experimentally observed uncertainties; triplicate measurements at each condition are recommended in future work to obtain directly measured standard deviations comparable with data from the literature.

**Table 9:** Estimated uncertainty budget for Cu(II) solution preparation and the principal analytical methods used in this study (manufacturer- or standard-specification-based; not an in-study replicate measurement).

Source of Uncertainty	Basis/Typical Specification	Relative Uncertainty (%)
Analytical balance (CuSO_4_·5H_2_O weighing, 3.929 g)	4-decimal balance, readability ± 0.0001 g	≈0.003
Stock solution volumetric flask (1000 mL)	Class A volumetric glassware (ISO 1042 tolerance ± 0.40 mL)	≈0.04
Serial dilution to working concentrations (10–500 mg L^−1^)	Class A pipettes/flasks, combined tolerance	≈0.3
CuSO_4_·5H_2_O reagent purity	Analytical reagent (AR) grade, manufacturer assay range ≈ 99–102%	≈0.9
Combined solution preparation uncertainty	Root-sum-of-squares of the four rows above	≈1.0
Temperature control (shaking bath/incubator)	Thermostatic control, 298, 303, 313 ± 1 K (Section 3.4.1, Section 3.4.2 and Section 3.4.3)	propagated into Table 6 (Van’t Hoff regression)
pH adjustment (0.1 M HCl/NaOH)	Typical combination pH–electrode accuracy	≈0.02 pH units
ICP–OES/AAS determination of residual Cu(II) (C_e_, C_t_)	Typical instrumental precision for aqueous Cu standards near the calibration range	≈2 (RSD)
CHNS elemental analysis	Manufacturer-specified instrument reproducibility	±0.1–0.3 wt% (absolute)
Combined uncertainty on single-determination C_e_/q_e_	Root-sum-of-squares of solution preparation and ICP–OES/AAS terms	≈2.2

## 4. Conclusions

Duck bone char, DKB, prepared at 300, 400, and 500 °C establishes a pyrolysis–structure–adsorption–regeneration relationship for a bone-derived Cu(II) adsorbent. Increasing pyrolysis temperature progressively removed and condensed the collagen–lipid matrix while preserving the calcium mineral scaffold of a constant crystallite size. Porosity evolved steadily with pyrolysis temperature; surface area and pore volume increased progressively from 300 to 500 °C, with no genuine collapse at 400 °C. Cu(II) capacity also increased steadily with temperature, but its area-normalized value was highest at 300 °C and essentially similar at 400 and 500 °C, showing that uptake depends more on the accessibility of apatite phosphate, hydroxyl, and Ca^2+^ sites than on surface area alone. Stretched exponential kinetic models and three- and four-parameter isotherm models described the data far better than pseudo-second-order and Langmuir models. Their exponents above unity indicated cooperative, multistep uptake. Spontaneous, strongly endothermic adsorption and points of zero charge of about pH 7–9.5 pointed to Ca^2+^–Cu^2+^ ion exchange, phosphate and hydroxyl complexation, and proposed surface nucleation of Cu-phosphate clusters as the dominant pathways (Cu(OH)_2_ precipitation not expected at the pH used), with electrostatic attraction and pore filling playing only minor roles. Pore development mainly accelerated the uptake rate, making DKB500 the fastest and highest-capacity char. After five HCl regeneration cycles, DKB retained 73.1–80.0% of its initial efficiency. The first-order decay constant decreased with pyrolysis temperature, so DKB500 was also the most durable. These results support duck bone as a low-cost, circular feedstock for Cu(II) remediation, pending a full life-cycle and cost assessment. Future work should confirm the binding mechanism by post-uptake XPS and XRD with Ca^2+^-release measurements, record measured reactor energy and yield for a primary life-cycle assessment, and test DKB500 in real industrial wastewater and continuous-flow systems.

## Figures and Tables

**Table 1:** Rietveld refinement results for duck bone char (DKB) produced at 300, 400, and 500 °C (Cu Kα, 10–80° 2θ; hydroxyapatite, P6_3_/m, and calcite, R-3c).

Parameter	300 °C	400 °C	500 °C
HAp a (Å)	9.4541(22)	9.4479(26)	9.4814(32)
HAp c (Å)	6.8865(16)	6.8787(21)	6.8688(25)
HAp cell volume (Å^3^)	533.0	531.8	534.8
Apparent size ⊥ c (nm)	7.4	7.6	7.6
Apparent size ∥ c (nm)	22.8	22.9	25.2
March–Dollase r [001]	1.108(7)	1.098(8)	1.136(11)
HAp (wt%)	98.2	99.1	98.9
Calcite (wt%)	1.8(2)	0.9(2)	1.1(2)
Rwp (%)	6.33	7.04	7.38
Rp (%)	4.99	5.49	5.77
Rexp (%)	5.23	5.68	5.84
χ^2^	1.46	1.54	1.59

**Table 2:** BET surface area and pore characteristics of duck bone char produced at different pyrolysis temperatures.

Property	Unit	DKB		
		300 °C	400 °C	500 °C
Specific surface area	m^2^ g^−1^	32.972	73.468	84.181
Total pore volume	cm^3^ g^−1^	0.101	0.203	0.269
Average pore diameter	nm	12.271	11.046	12.789

**Table 4:** Nonlinear kinetic model parameters and statistical fitting criteria at 300, 400, and 500 °C for duck bone char.

Model	Parameter/Statistic	DKB		
		300 °C	400 °C	500 °C
PFO q_t_ = q_e_[1 − exp(−k_1_t)] [25]	k_1_	0.04072	0.04179	0.04949
	q_e_	43.22	48.33	51.78
	SSE	39.51	22.47	21.99
	R^2^	0.9813	0.9911	0.9921
	Adj. R^2^	0.9790	0.9900	0.9912
	RMSE	2.222	1.676	1.658
	χ^2^	2.086	0.842	0.634
PSO q_t_ = (k_2_q_e_^2^t)/(1 + k_2_q_e_t) [26]	k_2_	0.0009540	0.0009060	0.001112
	q_e_	50.35	55.93	58.58
	SSE	103.7	85.44	94.98
	R^2^	0.9510	0.9663	0.9661
	Adj. R^2^	0.9449	0.9621	0.9618
	RMSE	3.600	3.268	3.446
	χ^2^	4.960	3.104	2.800
Avrami q_t_ = q_e_{1 − exp[−(k_av_t)^n^_^av^_]} [27]	k_av_	0.04156	0.04239	0.04930
	q_e_	42.27	47.55	51.17
	n_av_	1.480	1.296	1.254
	SSE	3.362	3.437	6.728
	R^2^	0.9984	0.9986	0.9976
	Adj. R^2^	0.9980	0.9983	0.9969
	RMSE	0.6930	0.7007	0.9804
	χ^2^	0.1326	0.1155	0.2345
DEM q_t_ = q_e_ − D_1_e^−k1t^ − (q_e_ − D_1_)e^−k2t^ [28]	q_e_	42.61	47.84	51.41
	D_1_	56.72	57.54	60.90
	k_1_	0.05506	0.05116	0.05883
	k_2_	13.43	12.41	19.19
	SSE	10.31	8.139	11.75
	R^2^	0.9951	0.9968	0.9958
	Adj. R^2^	0.9927	0.9952	0.9937
	RMSE	1.311	1.165	1.399
	χ^2^	0.4018	0.2473	0.3509
Weibull with lag time q_t_ = q_e_{1 − exp[−(k(t − t_0_))^m^]} [29]	q_e_	42.24	47.52	51.14
	k	0.03934	0.04103	0.04772
	m	1.579	1.347	1.301
	t_0_	−1.255	−0.7146	−0.6027
	SSE	3.088	3.189	6.388
	R^2^	0.9985	0.9987	0.9977
	Adj. R^2^	0.9978	0.9981	0.9966
	RMSE	0.7174	0.7291	1.032
	χ^2^	0.1087	0.1007	0.2176
Brouers–Weron–Sotolongo q_t_ = q_e_{1 − [1 + (*n* − 1)(t/τ)^a^]^−1/(n−1)^} [30]	q_e_	42.24	47.47	51.08
	τ	24.60	24.46	20.96
	α	1.443	1.250	1.211
	*n*	0.9434	0.9175	0.9261
	SSE	3.062	2.792	5.780
	R^2^	0.9986	0.9989	0.9979
	Adj. R^2^	0.9978	0.9983	0.9969
	RMSE	0.7144	0.6821	0.9815
	χ^2^	0.1176	0.09350	0.2060

**Table 5:** Nonlinear isotherm model parameters and statistical fitting criteria for DKB at 300, 400, and 500 °C.

Model	Parameter/Statistic	DKB		
		300 °C	400 °C	500 °C
Freundlich q_e_ = K_F_ C_e_^1/n^ [31]	K_F_	5.009	5.599	4.995
	1/*n*	0.4206	0.4065	0.4393
	SSE	241.3	266.7	321.3
	R^2^	0.8982	0.8945	0.8913
	Adj. R^2^	0.8836	0.8794	0.8758
	RMSE	5.872	6.172	6.775
	χ^2^	9.017	9.472	11.27
Langmuir q_e_ = q_m_K_L_C_e_/(1 + K_L_C_e_) [32]	q_m_	61.60	62.86	69.23
	K_L_	0.01510	0.01615	0.01395
	SSE	102.4	114.1	150.0
	R^2^	0.9568	0.9549	0.9493
	Adj. R^2^	0.9506	0.9484	0.9420
	RMSE	3.825	4.037	4.629
	χ^2^	4.233	4.446	5.782
Toth q_e_ = q_m_K_th_C_e_/[1 + (K_th_C_e_)^n^_^th^_]^1/n^_^th^_ [33]	q_m_	44.65	46.05	48.60
	Kth	0.01144	0.01177	0.01096
	nth	5.771	6.724	9.904
	SSE	4.933	3.198	10.11
	R^2^	0.9979	0.9987	0.9966
	Adj. R^2^	0.9972	0.9983	0.9954
	RMSE	0.9067	0.7301	1.298
	χ^2^	0.3378	0.2541	0.6290
Sips q_e_ = q_m_(K_S_C_e_)^n^_^s^_/[1 + (K_S_C_e_)^n^_^s^_] [34]	q_m_	46.61	48.13	50.99
	K_s_	2.14 × 10^−4^	2.15 × 10^−4^	1.05 × 10^−4^
	n_s_	2.243	2.264	2.409
	SSE	7.861	9.888	14.54
	R^2^	0.9967	0.9961	0.9951
	Adj. R^2^	0.9956	0.9948	0.9935
	RMSE	1.145	1.284	1.557
	χ^2^	0.5122	0.5283	0.8848
Fritz–Schlunder q_e_ = k_1_C_e_^a^/(1 + k_2_C_e_^b^) [35]	k_1_	0.05446	0.05470	0.02208
	a	1.701	1.727	1.968
	k_2_	3.12 × 10^−4^	3.24 × 10^−4^	1.74 × 10^−4^
	b	1.937	1.953	2.134
	SSE	1.350	2.346	9.038
	R^2^	0.9994	0.9991	0.9969
	Adj. R^2^	0.9991	0.9985	0.9951
	RMSE	0.5196	0.6850	1.344
	χ^2^	0.07610	0.09110	0.3420

**Table 6:** Langmuir isotherm and thermodynamic parameters for Cu(II) adsorption onto duck bone char produced at different pyrolysis temperatures.

Sample	Temp. (K)	K_L_ (L mg^−1^)	K_L_ (L mol^−1^)	ΔG° (kJ mol^−1^)	ΔH° (kJ mol^−1^)	ΔS° (kJ mol^−1^ K^−1^)
DKB (300 °C)	298	0.01500	953.2	−17.00 ± 0.45	71.45 ± 15.23	0.2977 ± 0.0500
	303	0.03200	2033	−19.19 ± 0.32		
	313	0.06300	4003	−21.59 ± 0.53		
DKB (400 °C)	298	0.01600	1017	−17.16 ± 0.30	80.04 ± 10.31	0.3268 ± 0.0339
	303	0.03300	2097	−19.27 ± 0.21		
	313	0.07800	4957	−22.14 ± 0.36		
DKB (500 °C)	298	0.01400	889.6	−16.82 ± 0.35	90.45 ± 12.12	0.3607 ± 0.0398
	303	0.03200	2033	−19.19 ± 0.25		
	313	0.08400	5338	−22.33 ± 0.42		

**Table 7:** Cycle-domain model parameters and statistical fitting criteria for Cu(II) removal of DKB during repeated adsorption–desorption cycles.

Model	Parameter	DKB 300 °C	DKB 400 °C	DKB 500 °C
M1 First-order E = E_0_e^−kn^	E_0_	1.011	1.011	1.011
	k (cycle^−1^)	0.0783	0.0674	0.0558
	SSE	6.74 × 10^−5^	9.18 × 10^−5^	1.19 × 10^−4^
	R^2^	0.9983	0.9971	0.9948
M2 Power-law E = E_0_n^−b^	E_0_	0.9509	0.9599	0.9683
	b	0.1807	0.1559	0.1291
	SSE	2.77 × 10^−3^	2.39 × 10^−3^	2.04 × 10^−3^
	R^2^	0.9303	0.9240	0.9117
M3 Avrami E = e^−(kn)^m^	m	1.073	1.091	1.118
	k (cycle^−1^)	0.0820	0.0722	0.0624
	SSE	3.34 × 10^−5^	4.91 × 10^−5^	5.55 × 10^−5^
	R^2^	0.9992	0.9984	0.9976
M4 Residual-activity E = E_i_ + (1 − E_i_)e^−kn^	E_i_	0	0	0
	k (cycle^−1^)	0.0752	0.0641	0.0526
	SSE	1.46 × 10^−4^	1.84 × 10^−4^	2.11 × 10^−4^
	R^2^	0.9963	0.9942	0.9909

Note: E = normalized Cu(II) removal efficiency; E_0_ = initial normalized efficiency; E_i_ = residual/asymptotic normalized efficiency; *n* = adsorption–desorption cycle number; k = cycle-domain decay constant; b = power-law decay exponent; m = Avrami shape exponent.

**Table 8:** Comparison of bone-derived adsorbents reported in the literature with DKB developed in the present study.

Bone-Derived Adsorbent	Target Pollutant	Preparation/Condition	Adsorption Performance	Regeneration/Key Finding	Ref.
Commercial bone char	Cu(II), Zn(II)	Batch adsorption, ~pH 5	Effective Cu(II)/Zn(II) uptake	HAp responsible for adsorption and Ca^2+^–metal ion exchange	[38]
Bone char	Cd(II)	Batch adsorption	0.57 mmol g^−1^	High Cd affinity; kinetic behavior evaluated using multiple models	[39]
Animal bone HAp (cow, pig, fish)	Cu(II)	Calcination; various temperatures	Nearly 100% Cu removal at pH > 7	Cu^2+^ substituted for Ca^2+^ in bone HAp	[40]
Bone char	Cu(II), Ni(II), Cd(II)	Batch adsorption	Cu: 1.093 mmol g^−1^; Cd: 0.760 mmol g^−1^; Ni: 0.453 mmol g^−1^ at pH_i_ 3	Affinity: Cu > Cd > Ni; ion exchange and apatite interactions	[41]
Chicken bone-derived char	Pb(II)	Chars from different chicken bone parts	Effective Pb sorption	Demonstrated influence of bone part/feedstock characteristics	[21]
Cow, chicken and pig bone char	Fluoride	Optimized charring	Cow bone char: 0.788 mg–F g^−1^–HAp	PSO kinetics and Langmuir equilibrium; surface area important	[42]
Bone char	Pb(II)	Batch adsorption	158.36 mg g^−1^ under reported optimum conditions	High Pb affinity; competitive adsorption with Cd/Ni/Cu investigated	[43]
H_2_O_2_-treated bone char	Cd(II)	H_2_O_2_ pretreatment + pyrolysis at 300 °C	228.73 mg g^−1^	Ion exchange, surface complexation and electrostatic attraction	[44]
Fish bone-derived natural HAp	Cu(II)	Alkaline heat treatment	28.97 mg g^−1^	PSO + Langmuir; spontaneous/endothermic adsorption; EDTA regeneration	[45]
Pig bone hydroxyapatite	Cu(II)	Natural HAp powder from pig–bone waste	Rapid Cu adsorption	Demonstrated direct valorization of pig bone HAp for Cu remediation	[18]
Cow bone char	Cu(II), Ni(II)	Bone char adsorption with nonlinear kinetic/isotherm analysis	Effective Cu/Ni removal; several kinetic and equilibrium models compared	Demonstrated importance of nonlinear model selection	[22]
Calcined bone char	Organic contaminant (tamoxifen)	Calcination	98.30 mg g^−1^	Langmuir + PSO; demonstrates broader pollutant-removal capability of bone char	[46]
DKB300–500	Cu(II)	Slow pyrolysis, 300–500 °C	Effective Cu(II) adsorption across temperature series	73.1–80.0% retained after 5 cycles; k = 0.056–0.078 cycle^−1^	This study

## Data Availability

Data are contained within the article. The original contributions presented in this study are included in the article. Further inquiries can be directed to the corresponding author.

## Abbreviations

The following abbreviations are used in this manuscript:

AC	Activated carbon
BET	Brunauer–Emmett–Teller
BJH	Barrett–Joyner–Halenda
BWS	Brouers–Weron–Sotolongo
CHNS	Carbon, hydrogen, nitrogen, and sulfur elemental analysis
Cu(II)	Divalent copper ion
DEM	Double-exponential model
DKB	Duck bone char
DKB300,DKB400, DKB500	Duck bone char pyrolyzed at 300, 400, and 500 °C
EDS	Energy-dispersive X-ray spectroscopy
FTIR	Fourier-transform infrared spectroscopy
HAp	Hydroxyapatite
ISO	International Organization for Standardization
IUPAC	International Union of Pure and Applied Chemistry
PFO	Pseudo-first-order
pH_PZC_	pH at the point of zero charge
PSO	Pseudo-second-order
RMSE	Root mean square error
SEM	Scanning electron microscopy
SSE	Sum of squared errors
XPS	X-ray photoelectron spectroscopy
XRD	X-ray diffraction
